# The Burden of Antimicrobial Resistant Bacteremia in Ontario: A Population-Wide Analysis of Attributable Mortality From 110 Pathogen-Antibiotic Combinations

**DOI:** 10.1093/cid/ciaf213

**Published:** 2025-05-13

**Authors:** Kevin A Brown, Daniel J Fridman, Gary E Garber, Jennie Johnstone, Bradley J Langford, Valerie Leung, Derek MacFadden, Samir N Patel, Kevin L Schwartz, Beate Sander, Nick Daneman

**Affiliations:** Dalla Lana School of Public Health, University of Toronto, Toronto, Ontario, Canada; Public Health Ontario, Toronto, Ontario, Canada; ICES, Toronto, Ontario, Canada; ICES, Toronto, Ontario, Canada; Department of Medicine, University of Ottawa, Ottawa, Ontario, Canada; Sinai Health, Toronto, Ontario, Canada; Department of Laboratory Medicine and Pathobiology, University of Toronto, Toronto, Ontario, Canada; Dalla Lana School of Public Health, University of Toronto, Toronto, Ontario, Canada; Public Health Ontario, Toronto, Ontario, Canada; Public Health Ontario, Toronto, Ontario, Canada; ICES, Toronto, Ontario, Canada; Department of Medicine, University of Ottawa, Ottawa, Ontario, Canada; Public Health Ontario, Toronto, Ontario, Canada; Department of Laboratory Medicine and Pathobiology, University of Toronto, Toronto, Ontario, Canada; Dalla Lana School of Public Health, University of Toronto, Toronto, Ontario, Canada; Public Health Ontario, Toronto, Ontario, Canada; ICES, Toronto, Ontario, Canada; Toronto General Hospital Research Institute, Toronto, Ontario, Canada; Institute of Health Policy, Management and Evaluation, University of Toronto, Toronto, Ontario, Canada; Public Health Ontario, Toronto, Ontario, Canada; ICES, Toronto, Ontario, Canada; Institute of Health Policy, Management and Evaluation, University of Toronto, Toronto, Ontario, Canada; Division of Infectious Diseases, Sunnybrook Research Institute, Toronto, Ontario, Canada

**Keywords:** antibiotic resistance, sepsis, methicillin-resistant staphylococcus aureus (MRSA), vancomycin-resistant enterococci (VRE), carbapenem-resistant Enterobacterales (CRE)

## Abstract

**Background:**

Reliable information on the burden of antimicrobial resistance (AMR) is necessary to confront the threat of antimicrobial resistance. We sought to examine the association between AMR and mortality across cultured bacterial bloodstream pathogens in the province of Ontario, Canada.

**Methods:**

We used linked microbiology data from 114 hospital, community, and public health laboratories to develop a positive bacterial blood culture episode cohort, between January 2017 and December 2021, for the population of Ontario, Canada (population 14.6 million). Antibiotics tested in >10% of cultures of a pathogen, with resistance 1%–99%, were eligible. We used separate proportional hazards models for each pathogen, to estimate the hazard ratio of 30-day mortality for each eligible antibiotic, adjusting for patient risk factors, and summarized results using mixed-effects meta-analysis.

**Results:**

We identified 83 962 bacteremia episodes, due to 30 pathogens, and 110 eligible pathogen-antibiotic combinations. The 30-day mortality was 17.1% (14 362/83 962). Unadjusted associations between resistance and 30-day mortality were substantially larger (hazard ratio [HR] = 1.47; 95% confidence interval [CI], 1.32–1.65) than adjusted associations accounting for age, sex, healthcare exposures, comorbidities, and co-resistance (HR = 1.10; 95% CI, 1.07–1.16). Associations were larger for antibiotics commonly used for empiric treatment (HR = 1.18; 95% CI, 1.10–1.26).

**Conclusions:**

We found that antimicrobial resistance was associated with a 10% relative increase in the risk of mortality among patients with bacteremia, and 1.2 AMR attributable deaths per 100 000 population per year in Ontario, Canada. Comprehensive risk adjustment is necessary for understanding the impact of AMR bacteremia on patient outcomes.

## Teaser

We sought to measure the association between antimicrobial resistanceand mortality among patients with bacterial bloodstream pathogens. Among 83 962 bacteremia episodes due to 30 separate pathogens, we identified a 10% relative increase in the risk of 30-day mortality.

## BACKGROUND

Reliable information on the burden of antimicrobial resistance (AMR) is necessary to confront the threat of antimicrobial resistance. The Antimicrobial Resistance Collaborators recently reported the most comprehensive global estimate of the burden of AMR across 23 pathogens, 88 pathogen-antibiotic combinations in 204 countries [1,2]. These estimates were contingent on comprehensive information of the geographic distribution of incident AMR infections, as well as the attributable mortality due to resistant relative to susceptible pathogens but were based on many studies that did not adjust for patient comorbidities and degree of healthcare exposure [3,4].

Few jurisdictions have the ability to comprehensively examine adjusted estimates of AMR attributable mortality, because microbiology data are often siloed in smaller local hospital data systems and cannot be linked to important demographic, clinical, and outcomes data [3]. Inadequate adjustment for patient comorbidities and healthcare exposures may lead to overestimation of the impacts of AMR because patients with resistant infections tend to be older, frailer, and have extensive prior exposures to healthcare environments [5]. Comprehensive linked microbiology data are available for the population of Ontario, Canada (14.6 million, 2019), through a province-wide database (Ontario Laboratories Information System [OLIS]), and has been previously used for studies of bacteremia [6] and audit and feedback [7].

In this study, examined the association between AMR and mortality across all cultured bacterial bloodstream pathogens. We hypothesized that patients infected with more resistant pathogens, compared to patients with fully susceptible versions of the same pathogen, would be at increased risk of 30-day mortality.

## METHODS

### Data Sources

The study was made possible by the availability of province-wide microbiology data from OLIS, combining data from 114 hospital, community, and public health laboratories into a single repository. OLIS is linkable to administrative datasets from Ontario's universal healthcare system via a unique and confidential identifier held at ICES. ICES is an independent, nonprofit research institute whose legal status under Ontario's health information privacy law allows it to collect and analyze healthcare and demographic data for health system evaluation and improvement. The linked databases for this study included hospital information from the Discharge Abstract Database, emergency department data from the National Ambulatory Care Reporting System, physician claims data from the Ontario Health Insurance Plan, and vital statistics and demographic data from the Registered Persons Database. Organism and antibiotic names are reported using International Organization for Standardization conventions with the assistance of the R AMR package [8].

### Population

We developed a cohort based on positive blood culture episodes among Ontario residents from January 2017 to December 2021. All blood cultures with the same organism within 7 days of a first positive culture were considered part of the same episode. The index date of the episode was the date of the first positive culture. A single person could have multiple episodes. Episodes with polymicrobial cultures were excluded.

We restricted our analysis to pathogens responsible for at least 150 positive bloodstream infection episodes and at least 20 associated deaths across the study period. To ensure inclusion of commonly tested antibiotics and accurate imputation of resistance, for each organism, we selected antibiotics for which susceptibility results were reported in at least 10% of episodes, with a resistance proportion between 1% to 99%, and that did not share a Pearson correlation of greater than 0.9 with another form of antimicrobial resistance. Finally, we excluded pathogen–antibiotic combinations for which there were no deaths associated.

### Exposure—Antimicrobial Susceptibility

Because there was variability in testing and reporting of antimicrobial susceptibility results for isolates across laboratories, we applied imputation rules for isolates with missing resistance results. First, we applied rules-based imputation to account for intrinsic susceptibility or resistance patterns, as well as those that can be extrapolated from known susceptibility results (see Supplement 1.1). For those still missing after rules-based imputation, we developed separate imputation models for each pathogen–antibiotic using logistic regression that included, as covariates: age, sex, location at time of culture collection, and the results (each coded as susceptible, not susceptible, or missing) of all other antibiotics; an approach paralleling prior studies [6,9]. As is customary for imputation, we calibrated these models using the nonmissing pathogen–antibiotic susceptibilities and then used the models to predict the probability of resistance for missing values.

In addition to susceptibilities, a separate covariable, termed treatment relevance, was measured to capture whether a given antimicrobial was considered important for empiric treatment in Ontario based on adjudication by 2 study authors (K. S. and D. M.) that were blinded to the study outcomes. Each adjudicator rated each antibiotic's treatment relevance for each pathogen, on a 3-point scale (low = 0, moderate = 0.5, high = 1), and the average of the 2 ratings was taken, yielding a 5-point scale (0 = low, 0.25 = moderate-low, 0.5 = moderate, 0.75 = moderate-high, 1 = high).

### Covariables

Patient covariables included age, sex, setting at the time of blood culture draw (community, hospital ward, intensive care unit [ICU]), total days spent in hospital in prior 12 months, total days spent in long-term care in prior 12 months, and total physician visits in prior 12 months. We coded 19 comorbidities, based on validated algorithms [10, 11], using International Classification of Diseases, 10th revision–coded hospital and emergency unit discharges in the prior 24 months. Because small sample sizes for certain pathogens for specific comorbidities caused convergence issues, we included 3 of the most important individual comorbidities (based on prevalence and highest contribution to risk of 30-day mortality across the pathogens) as individual risk factors (cancer, renal disease, congestive heart failure) and created a variable representing a comorbidity count for the remaining 16 (osteoarthritis, cardiac arrhythmia, mood disorders, mental health disorders, osteoporosis, coronary artery disease, myocardial infarction, asthma, chronic obstructive pulmonary disease, dementia, diabetes mellitus, hypertension, rheumatoid arthritis, chronic liver disease, stroke, and immune suppressive conditions).

### Outcomes

Our primary outcome was 30-day mortality, defined as deaths within 30 days of the index positive blood culture date, based on Ontario's population registry. A single patient could have multiple episodes. If a patient had a subsequent episode within the 30-day window, the initial episode (and follow-up) was considered censored when a new episode (and follow-up) started.

### Pathogen-Specific Analyses

For each pathogen, we fitted Weibull proportional hazard regressions to assess the association between AMR and death within 30 days of the index blood culture. For Weibull regression, the outcome was the number of days elapsed from positive blood culture to death or censoring, whichever came first. We estimated 4 sets of hazard ratios for each pathogen–antibiotic:

- First, we measured unadjusted hazard ratios, using separate models for each antibiotic, that included the given antibiotic as the only covariable.
- Second, we measured patient-characteristic adjusted hazard ratios that were not adjusted for co-resistance, using separate models for each antibiotic, and that included the given antibiotic in addition to the patient covariables.
- Third, we measured co-resistance adjusted hazard ratios, that were adjusted for co-resistance but not patient characteristics, using a model that included all eligible antibiotics but no patient covariables.
- Fourth, we measured doubly adjusted hazard ratios, that were adjusted for co-resistance and patient characteristics, using a model that included all eligible antibiotics as well as the patient covariables.

### Pan-Pathogen Analyses

To summarize average resistance hazard ratios across all pathogen–antibiotic combinations, we used random effects meta-analysis separately for each of the 4 models here. For these analyses, we used a dataset with a single row for each pathogen–antibiotic combination and 4 separate pairs of variables corresponding to the log hazard ratio coefficients and standard errors.

In addition, we measured robust doubly adjusted hazard ratios for each pathogen–antibiotic, based on a multivariate mixed effects meta-regression model. Commonalities across the 30 pathogens (eg, a strong positive hazard ratio for increasing age) mean that all of the original parameters from the 30 pathogen-specific models can be more robustly estimated using such a meta-regression model [12], yielding partially pooled estimates [13, 14]. For this analysis, we used a dataset with all log hazard ratios from the 30 pathogen-specific models and a separate covariance matrix dataset (see supplement 1.5 for annotated code, and supplement 2 for data) [12]. The outcome variable in the meta-regression was the log hazard ratio. The fixed effects predictor variables were (1) a categorical variable representing the coefficient type (scale, intercept, sex, age, 11 risk factors, antibiotic) and (2) treatment relevance was included as a linear covariable; there were also random intercepts with standard deviations estimated separately for each coefficient type. Based on this meta-regression, we then extracted the resultant robust, or partially pooled, pathogen-specific parameter estimates for each of the 110 pathogen–antibiotic combinations. As an additional sensitivity analysis, we also considered the treatment relevance variable as a 5-level categorical covariable. All meta-analysis and meta-regression models were estimated using the brms package in R [15].

### Postestimation of Absolute Incidence

Because the robust doubly adjusted estimates were relative measures of AMR effects, we used marginal standardization [16] to estimate the absolute incidence of mortality for isolates with the absence of resistance (ie, for isolates completely susceptible), compared to isolates that had the resistance patterns and prevalences observed in the data. Marginal standardization was used to estimate absolute incidence for each pathogen–antibiotic pair, for each organism, and overall. We reported both absolute incidence and incidence rates based on Ontario's population of 14.6 million in 2019.

### Ethics

This research was reviewed and approved by the research ethics board of Public Health Ontario (ERB # 2018-012.01).

## RESULTS

**Figure 1.**
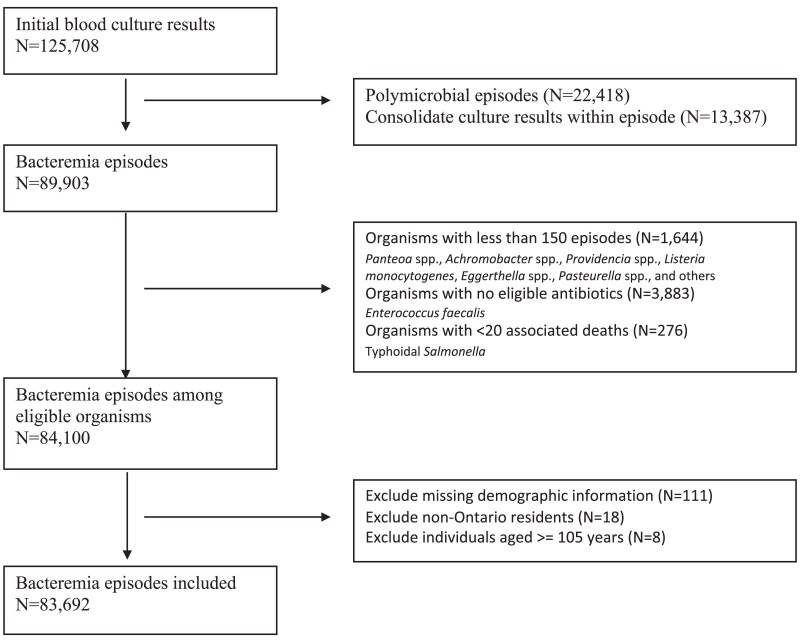
Flow diagram for inclusion of patient bacteremia episodes, between 2017 to 2021.

After exclusions, we identified 83 962 bacteremia episodes, due to 30 pathogens (Figure 1). Episodes with typhoidal *Salmonella* subspecies were excluded because of no associated deaths, and episodes with *Enterococcus faecalis* were excluded because the prevalence of resistance for the only commonly tested antibiotic (ampicillin) was less than 1%. Of the remaining episodes, 45 626 (54.3%) were caused by 14 gram-negative pathogens and 38 336 (45.7%) were caused by 16 gram-positive pathogens. The most common pathogens were *Escherichia coli* (25 928, 30.8%), *Staphylococcus aureus* (14 854, 17.6%) and *Klebsiella* spp. (8382, 10.0%). The median patient age was 70 years (interquartile range: 56–81 years) and 54.9% (46 210) were male. The median inpatient duration of stay at the time of sample collection was 4 days (interquartile range: 0–20).

### Outcomes

A total of 17.1% (14 362/83 962) of patients died within 30 days of bacteremia diagnosis (Table 1). Mortality was 12.5% (3229/25 928) among those with *E. coli* bacteremia, 22.0% with *S. aureus* bacteremia (3269/14 854), and was highest with *Enterococcus faecium* bacteremia at 33.3% (578/1738).

**Table 1.** Characteristics of Patients With Bacteremia Due to 30 Pathogen Types (N = 84 238) in Ontario Between 2017 and 2021

		Male Sex	Age	ICU	Inpatient-days	Cancer	CHF	Renal Disease	30-d Mortality
	N	N (%)	Median (IQR)	N (%)	Median (IQR)	N (%)	N (%)	N (%)	N (%)
Total	83 962	46 210 (54.9%)	70 (56–81)	12 588 (14.9%)	4 (0–20)	46 074 (54.9%)	20 853 (24.8%)	28 451 (33.9%)	14 362 (17.1%)
**Gram-negative bacteria**									
*Enterobacterales*									
*Escherichia coli*	25 928	11 783 (45.4%)	74 (62–83)	2698 (10.4%)	1 (0–12)	15 145 (58.4%)	5696 (22.0%)	7937 (30.6%)	3229 (12.5%)
*Klebsiella* spp.	8382	4982 (59.4%)	71 (60–81)	1198 (14.3%)	8 (0–27)	5233 (62.4%)	1934 (23.1%)	3095 (36.9%)	1443 (17.2%)
*Enterobacter* spp.	1975	1240 (62.8%)	69 (57–78)	405 (20.5%)	13 (3–35)	1250 (63.3%)	463 (23.4%)	760 (38.5%)	334 (16.9%)
*Proteus* spp.	1847	1051 (56.9%)	77 (67–86)	224 (12.1%)	4 (0–23)	1069 (57.9%)	494 (26.7%)	729 (39.5%)	380 (20.6%)
*Serratia* spp.	925	618 (66.8%)	68 (58–78)	218 (23.6%)	12 (2–33)	497 (53.7%)	316 (34.2%)	413 (44.6%)	213 (23.0%)
*Citrobacter* spp.	622	425 (68.3%)	72 (61–81)	109 (17.5%)	8 (0–25)	391 (62.9%)	155 (24.9%)	242 (38.9%)	115 (18.5%)
*Salmonella,* nontyphoidal	425	240 (56.5%)	54 (27–70)	33 (7.8%)	0 (0–1)	156 (36.7%)	44 (10.4%)	76 (17.9%)	24 (5.6%)
*Morganella* spp.	285	186 (65.3%)	75 (64–85)	33 (11.6%)	3 (0–16)	157 (55.1%)	91 (31.9%)	107 (37.5%)	66 (23.2%)
Nonfermenters									
*Pseudomonas aeruginosa*	3082	1931 (62.7%)	72 (61–81)	633 (20.5%)	16 (3–42)	2103 (68.2%)	934 (30.3%)	1344 (43.6%)	794 (25.8%)
*Acinetobacter* spp.	573	314 (54.8%)	61 (45–73)	100 (17.5%)	8 (1–25)	271 (47.3%)	118 (20.6%)	179 (31.2%)	84 (14.7%)
*Stenotrophomonas* spp.	290	167 (57.6%)	58 (41–68)	47 (16.2%)	26 (12–58)	164 (56.6%)	50 (17.2%)	120 (41.4%)	69 (23.8%)
*Pseudomonas* spp., other	267	146 (54.7%)	62 (40–78)	34 (12.7%)	13 (2–40)	152 (56.9%)	70 (26.2%)	97 (36.3%)	48 (18.0%)
Other									
*Hemophilus* spp.	548	265 (48.4%)	64 (42–78)	103 (18.8%)	1 (0–6)	260 (47.4%)	117 (21.4%)	123 (22.4%)	87 (15.9%)
*Bacteroides* spp.	477	272 (57.0%)	71 (58–82)	63 (13.2%)	6 (0–19)	294 (61.6%)	107 (22.4%)	162 (34.0%)	122 (25.6%)
**Gram-positive bacteria**									
*Staphylococcus*									
*Staphylococcus aureus*	14 854	9122 (61.4%)	65 (50–78)	2240 (15.1%)	5 (0–21)	6937 (46.7%)	4161 (28.0%)	5629 (37.9%)	3269 (22.0%)
*Staphylococcus* spp., other	5240	2889 (55.1%)	66 (52–78)	1359 (25.9%)	8 (0–31)	2549 (48.6%)	1420 (27.1%)	1788 (34.1%)	948 (18.1%)
*Staphylococcus epidermidis*	3693	2096 (56.8%)	66 (50–77)	778 (21.1%)	11 (1–31)	1809 (49.0%)	1089 (29.5%)	1451 (39.3%)	662 (17.9%)
*Staphylococcus lugdunensis*	419	262 (62.5%)	67 (56–78)	55 (13.1%)	2 (0–12)	231 (55.1%)	112 (26.7%)	154 (36.8%)	79 (18.9%)
*Streptococcus*									
*Streptococcus* spp., other	2993	1800 (60.1%)	68 (55–80)	381 (12.7%)	1 (0–10)	1588 (53.1%)	736 (24.6%)	749 (25.0%)	447 (14.9%)
*Streptococcus pneumoniae*	2332	1314 (56.3%)	62 (49–73)	490 (21.0%)	0 (0–4)	1040 (44.6%)	320 (13.7%)	415 (17.8%)	357 (15.3%)
*Streptococcus pyogenes*	1612	891 (55.3%)	57 (40–71)	314 (19.5%)	0 (0–7)	575 (35.7%)	316 (19.6%)	369 (22.9%)	207 (12.8%)
*Streptococcus group B*	1606	861 (53.6%)	68 (54–80)	177 (11.0%)	1 (0–7)	833 (51.9%)	454 (28.3%)	493 (30.7%)	206 (12.8%)
*Streptococcus group C/G*	1299	782 (60.2%)	73 (61–83)	153 (11.8%)	1 (0–9)	721 (55.5%)	475 (36.6%)	416 (32.0%)	184 (14.2%)
*Streptococcus viridians* spp.	943	521 (55.2%)	65 (52–77)	123 (13.0%)	6 (0–23)	574 (60.9%)	231 (24.5%)	265 (28.1%)	108 (11.5%)
*Streptococcus mitis*	612	376 (61.4%)	65 (43–78)	51 (8.3%)	1 (0–14)	331 (54.1%)	155 (25.3%)	145 (23.7%)	80 (13.1%)
*Enterococcus*									
*Enterococcus faecium*	1738	1012 (58.2%)	67 (57–77)	442 (25.4%)	32 (16–61)	1158 (66.6%)	551 (31.7%)	854 (49.1%)	578 (33.3%)
*Enterococcus* spp., other^a^	299	162 (54.2%)	69 (58–80)	55 (18.4%)	14 (2–32)	186 (62.2%)	79 (26.4%)	125 (41.8%)	76 (25.4%)
Other									
*Clostridium* spp.	341	175 (51.3%)	71 (58–82)	45 (13.2%)	9 (1–22)	226 (66.3%)	79 (23.2%)	103 (30.2%)	99 (29.0%)
*Actinomyces* spp.	203	99 (48.8%)	64 (45–80)	11 (5.4%)	2 (0–14)	94 (46.3%)	43 (21.2%)	57 (28.1%)	33 (16.3%)
*Granulicatella* spp.	152	91 (59.9%)	63 (42–79)	16 (10.5%)	4 (0–18)	80 (52.6%)	43 (28.3%)	52–56 (34–37%)	21 (13.8%)

Abbreviations: CHF, congestive heart failure; ICU, intensive care unit; IQR, interquartile range.

^a^This group excludes *Enterococcus faecalis*.

### Antibiotic Resistance

Across the 30 pathogens, susceptibilities for 110 pathogen–antibiotic combinations (average of 3.7 per pathogen, min = 1, max = 8) were eligible based on being measured in >10% of isolates and exhibiting resistance prevalence between 1% and 99% (Table 2). Among *E. coli* episodes, ceftriaxone resistance was 13.0% (3371/25 928). Among *S. aureus* episodes, cefazolin resistance (indicating methicillin-resistant *S. aureus* [MRSA]) was 17.0% (2530/14 854). Among *E. faecium* episodes, vancomycin resistance was 24.1% (419/1738).

**Table 2.** Prevalence (%) of Nonresistance for 30 Pathogens and 17 Antibiotics With >10% Testing (N = 110 Antibiotic Susceptibilities) Among Patients With Bacteremia in Ontario Between 2017 and 2021

Antibiotic Class		AMI	AMI	PEN	PEN	BLBI	BLBI	CG1	CG3	CG3	CP	CP	FL	FL	LI	ML	SU	X
Antibiotic name		GEN	TOB	AMP	BPEN	AMC	TZP	CZO	CAZ	CRO	ETP	MEM	CIP	LVX	CLI	ERY	SXT	VAN
	**N**	**…**	**…**	**…**	**…**	**…**	**…**	**…**	**…**	**…**	**…**	**…**	**…**	**…**	**…**	**…**	**…**	**…**
**Gram-negative bacteria**																		
*Enterobacterales*																		
*Escherichia coli*	25 928	90	…	48	…	67	84	59	…	87	…	…	70	…	…	…	73	…
*Klebsiella* spp.	8382	96	…	…	…	80	87	65	…	89	…	…	89	…	…	…	89	…
*Enterobacter* spp.	1975	96	…	…	…	…	36	…	…	41	65	99	93	…	…	…	90	…
*Proteus* spp.	1847	92	…	74	…	86	98	62	…	96	…	…	86	…	…	…	85	…
*Serratia* spp.	925	…	…	…	…	…	37	…	…	41	97	…	96	…	…	…	…	…
*Citrobacter* spp.	622	97	…	…	…	…	48	6	…	39	98	…	95	…	…	…	92	…
*Salmonella,* nontyphoidal	425	…	…	91	…	…	…	…	…	…	…	…	65	…	…	…	…	…
*Morganella* spp.	285	93	…	…	…	…	68	…	…	46	…	…	89	…	…	…	84	…
*Nonfermenters*	*…*	*…*	*…*	*…*	*…*	*…*	*…*	*…*	*…*	*…*	*…*	*…*	*…*	*…*	*…*	*…*	*…*	*…*
*Pseudomonas aeruginosa*	3082	…	99	…	…	…	89	…	89	…	…	79	88	…	…	…	…	…
*Acinetobacter* spp.	573	96	…	…	…	…	80	…	85	…	…	96	93	…	…	…	94	…
*Stenotrophomonas* spp.	290	…	…	…	…	…	…	…	…	…	…	…	…	88	…	…	…	…
*Pseudomonas* spp., other	267	…	…	…	…	…	91	…	…	…	…	83	…	…	…	…	51	…
*Other*	*…*	*…*	*…*	*…*	*…*	*…*	*…*	*…*	*…*	*…*	*…*	*…*	*…*	*…*	*…*	*…*	*…*	*…*
*Hemophilus* spp.	548	…	…	81	…	…	…	…	…	…	…	…	…	…	…	…	…	…
*Bacteroides* spp.	477	…	…	…	…	…	78	…	…	…	…	…	…	…	54	…	…	…
**Gram-positive bacteria**																		
*Staphylococcus*																		
*Staphylococcus aureus*	14 854	…	…	…	4	…	…	83	…	…	…	…	…	…	32	72	97	…
*Staphylococcus* spp., other	5240	…	…	…	8	…	…	44	…	…	…	…	…	…	47	49	69	…
*Staphylococcus epidermidis*	3693	…	…	…	6	…	…	34	…	…	…	…	…	…	44	42	56	…
*Staphylococcus lugdunensis*	419	…	…	…	28	…	…	90	…	…	…	…	…	…	…	…	…	…
*Streptococcus*	…	…	…	…	…	…	…	…	…	…	…	…	…	…	…	…	…	…
*Streptococcus* spp., other	2993	…	…	…	86	…	…	…	…	98	…	…	…	…	79	…	…	…
*Streptococcus pneumoniae*	2332	…	…	…	95	…	…	…	…	…	…	…	…	…	92	82	66	…
*Streptococcus pyogenes*	1612	…	…	…	…	…	…	…	…	…	…	…	…	…	85	86	…	…
*Streptococcus group B*	1606	…	…	…	…	…	…	…	…	…	…	…	…	…	57	58	…	…
*Streptococcus group C/G*	1299	…	…	…	…	…	…	…	…	…	…	…	…	…	81	81	…	…
*Streptococcus viridians* spp.	943	…	…	…	66	…	…	…	…	97	…	…	…	…	…	…	…	…
*Streptococcus mitis*	612	…	…	…	75	…	…	…	…	96	…	…	…	…	…	…	…	…
*Enterococcus*	*…*	*…*	*…*	*…*	*…*	*…*	*…*	*…*	*…*	*…*	*…*	*…*	*…*	*…*	*…*	*…*	*…*	*…*
*Enterococcus faecium*	1738	…	…	11	…	…	…	…	…	…	…	…	…	…	…	…	…	76
*Enterococcus* spp., other	299	…	…	89	…	…	…	…	…	…	…	…	…	…	…	…	…	48
Other																		
*Clostridium* spp.	341	…	…	…	80	…	98	…	…	…	…	…	…	…	66	…	…	…
*Actinomyces* spp.	203	…	…	…	…	…	…	…	…	…	…	…	…	…	84	…	…	…
*Granulicatella* spp.	152	…	…	…	56	…	…	…	…	59	…	…	…	…	…	…	…	…

Cells with darker shading have higher prevalence of resistance.

Abbreviations: AMC, amoxicillin/clavulanic acid; AMI, aminoglycosides; AMP, ampicillin; BLBI, β-lactam beta-lactamase inhibitor combination; BPEN, benzylpenicillin; CAZ, ceftazidime, CG1, first-generation cephalosporins; CG3, third-generation cephalosporins; CIP, ciprofloxacin; CLI, clindamycin; CP, carbapenems; CRO, ceftriaxone; CZO, cefazolin; ERY, erythromycin; ETP, ertapenem; FL, fluoroquinolones; GEN, gentamicin; LI, lincosamides, LVX = levofloxacin; MEM, meropenem; ML, macrolides; PEN, penicillins; SU, sulfonamides; SXT, trimethoprim/sulfamethoxazole; TOB, tobramycin; TZP, piperacillin/tazobactam; VAN, vancomycin; X, other.

### Treatment Relevance

Across the 110 pathogen–antibiotic combinations, agreement between the reviewers on treatment relevance was moderate (Pearson correlation = 0.52, weighted kappa = 0.30). The average treatment relevance across the 110 pathogen–antibiotic pairs was between moderate and moderate-high (0.64; low = 11%, moderate-low = 17%, moderate = 14%, moderate-high = 23%, high = 36%). A complete listing of the treatment relevance ratings is provided in Supplement 1.2.

### Association Between Resistance and 30-Day Mortality

**Figure 2.**
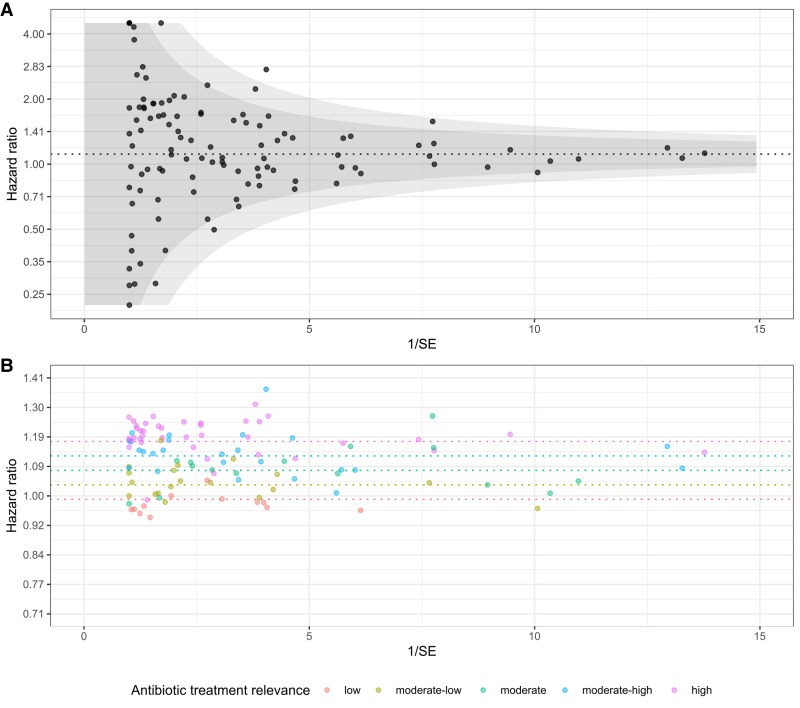
Estimated hazard ratios (N = 110 pathogen–antibiotic combinations) for association between antimicrobial resistance and 30-d mortality, among bacterial pathogens (N = 30), and across 17 antibiotics. The top panel (*A*) depicts the hazard ratios that were adjusted for patient characteristics and co-resistance, but subject to substantial sampling variability, particularly for uncommon pathogens and antibiotics. The dotted line is the average impact of resistance. The bottom panel (*B*) depicts the hazard ratios, after the pan-pathogen meta-regression model was used to stabilize the hazard ratio estimates. The dotted lines represent the average hazard ratios for antibiotics with low, moderate-low, moderate, moderate-high, and high treatment relevance. Abbreviation: SE, standard error.

Across the 110 pathogen–antibiotic combinations studied, the average unadjusted association for a given form of resistance was to increase risk of death by 47% (average hazard ratio [HR] = 1.47; 95% confidence interval [CI], 1.32–1.65). Adjustment for patient covariables alone, but not co-resistance, yielded lower average estimates (average HR = 1.30; 95% CI, 1.24–1.36). Adjustment for co-resistance that accounted for the fact that patients with 1 form of resistance were more likely to have other forms of resistance, were also lower relative to the unadjusted association (average HR = 1.16; 95% CI, 1.10–1.24). Adjustment for both patient covariables and co-resistance, yielded estimates that were further attenuated (HR = 1.10; 95% CI, 1.07–1.16). Estimated adjusted associations among rarer forms of resistance in rarer organisms were subject to greater sampling variability (Figure 2*A*).

Meta-regression of the 110 resistance effects (Figure 2*B* and Table 3), based on the linear treatment relevance covariable, suggested important associations were present for antibiotics with high treatment relevance (HR = 1.18; 95% CI, 1.10–1.26), but not those with low treatment relevance (HR = 0.99; 95% CI, 0.89–1.10). Based on this model, the estimated association between cefazolin resistance and 30-day mortality among *S. aureus* episodes (MRSA) was 1.14 (95% CI, 0.95–1.37), the estimated association between vancomycin resistance and 30-day mortality among *E. faecium* episodes (vancomycin-resistant enterococci) was 1.14 (95% CI, 0.85–1.54) and estimated association between ceftriaxone resistance among Enterobacterales was 1.17 (95% CI, 0.80–1.71) for *E. coli*, 1.26 (95% CI, 0.76–2.11) for *Klebsiella* spp., and 1.14 (95% CI, 0.61–2.16) for *Enterobacter* spp. Considering treatment relevance as a categorical variable, supported the initial linear covariable approach (low: HR = 0.98; 95% CI, .79–1.21; moderate-low: HR = 1.04; 95% CI, .89–1.22; moderate: HR = 1.10; 95% CI, .99–1.23; moderate-high: HR = 1.10; 95% CI, 1.00–1.22; high HR = 1.19; 95% CI, 1.09–1.30). Fixed-effects estimates of adjustment covariates in order of decreasing statistical importance were: age, ICU status, renal disease, congestive heart failure, long-term care days, inpatient days, cancer, and comorbidity count; hospital ward setting, sex, and primary care visits were not statistically significant.

**Table 3.** Hazard Ratios of Association Between Resistance and 30-Day Mortality, for 30 Pathogens and 17 Antibiotics (N = 110 Pathogen–Antibiotic Combinations) Among Patients With Bacteremia in Ontario Between 2017 and 2021

Antibiotic Class		AMI	AMI	PEN	PEN	BLBI	BLBI	CG1	CG3	CG3	CP	CP	FL	FL	LI	ML	SU	X
Antibiotic name		GEN	TOB	AMP	BPEN	AMC	TZP	CZO	CAZ	CRO	ETP	MEM	CIP	LVX	CLI	ERY	SXT	VAN
	**N**	…	…	…	…	…	…	…	…	…	…	…	…	…	…	…	…	…
**Gram-negative bacteria**																		
*Enterobacterales*																		
*Escherichia coli*	25 928	0.96	…	1.01	…	1.01	1.18	1.04	…	1.17	…	…	1.16	…	…	…	1.08	…
*Klebsiella* spp.	8382	1.07	…	…	…	1.19	1.12	1.03	…	1.26	…	…	1.08	…	…	…	1.08	…
*Enterobacter* spp.	1975	1.04	…	…	…	…	1.2	…	…	1.14	1.25	1.26	1.13	…	…	…	1.1	…
*Proteus* spp.	1847	1.04	…	1.11	…	1.07	1.23	1.07	…	1.21	…	…	1.11	…	…	…	1.05	…
*Serratia* spp.	925	…	…	…	…	…	1.19	…	…	1.23	1.25	…	1.14	…	…	…	…	…
*Citrobacter* spp.	622	1	…	…	…	…	1.18	1.09	…	1.2	1.19	…	1.17	…	…	…	1.09	…
*Salmonella,* nontyphoidal	425	…	…	0.98	…	…	…	…	…	…	…	…	0.99	…	…	…	…	…
*Morganella* spp.	285	1.04	…	…	…	…	1.24	…	…	1.21	…	…	1.14	…	…	…	1.13	…
Nonfermenters	…	…	…	…	…	…	…	…	…	…	…	…	…	…	…	…	…	…
*Pseudomonas aeruginosa*	3082	…	1.08	…	…	…	1.19	…	1.31	…	…	1.24	1.05	…	…	…	…	…
*Acinetobacter* spp.	573	1.07	…	…	…	…	1.19	…	1.22	…	…	1.26	1.18	…	…	…	1.2	…
*Stenotrophomonas* spp.	290	…	…	…	…	…	…	…	…	…	…	…	…	1.18	…	…	…	…
*Pseudomonas* spp., other	267	…	…	…	…	…	1.23	…	…	…	…	…	1.14	…	…	…	1.09	…
Other																		
*Hemophilus* spp.	548	…	…	1.1	…	…	…	…	…	…	…	…	…	…	…	…	…	…
*Bacteroides* spp.	477	…	…	…	…	…	1.19	…	…	…	…	…	…	…	1.11	…	…	…
**Gram-positive bacteria**																		
*Staphylococcus*																		
*Staphylococcus aureus*	14 854	…	…	…	0.97	…	…	1.14	…	…	…	…	…	…	1.04	0.96	1.08	…
*Staphylococcus* spp., other	5240	…	…	…	0.99	…	…	1.2	…	…	…	…	…	…	0.99	0.98	1.26	…
*Staphylococcus epidermidis*	3693	…	…	…	1	…	…	1.15	…	…	…	…	…	…	1.02	0.98	1.16	…
*Staphylococcus lugdunensis*	419	…	…	…	0.96	…	…	1.18	…	…	…	…	…	…	…	…	…	…
*Streptococcus*	…	…	…	…	…	…	…	…	…	…	…	…	…	…	…	…	…	…
*Streptococcus* spp., other	2993	…	…	…	1.13	…	…	…	…	1.17	…	…	…	…	0.98	…	…	…
*Streptococcus pneumoniae*	2332	…	…	…	1.24	…	…	…	…	…	…	…	…	…	0.96	1.03	1.01	…
*Streptococcus pyogenes*	1612	…	…	…	…	…	…	…	…	…	…	…	…	…	0.99	0.95	…	…
*Streptococcus group B*	1606	…	…	…	…	…	…	…	…	…	…	…	…	…	1.07	1.05	…	…
*Streptococcus group C/G*	1299	…	…	…	…	…	…	…	…	…	…	…	…	…	1	0.94	…	…
*Streptococcus viridians* spp.	943	…	…	…	1.07	…	…	…	…	1.15	…	…	…	…	…	…	…	…
*Streptococcus mitis*	612	…	…	…	1.15	…	…	…	…	1.22	…	…	…	…	…	…	…	…
*Enterococcus*	…	…	…	…	…	…	…	…	…	…	…	…	…	…	…	…	…	…
*Enterococcus faecium*	1738	…	…	1.37	…	…	…	…	…	…	…	…	…	…	…	…	…	1.14
*Enterococcus* spp., other*	299	…	…	1.24	…	…	…	…	…	…	…	…	…	…	…	…	…	1.11
Other																		
*Clostridium* spp.	341	…	…	…	1.11	…	1.18	…	…	…	…	…	…	…	1.09	…	…	…
*Actinomyces* spp.	203	…	…	…	…	…	…	…	…	…	…	…	…	…	0.97	…	…	…
*Granulicatella* spp.	152	…	…	…	1.18	…	…	…	…	1.19	…	…	…	…	…	…	…	…

Estimates are based on separate organism-specific models adjusted for patient characteristics and co-resistance, and subsequent meta-regression. Confidence intervals included in Supplement 1.2. Cells with darker shading have higher hazards ratios.

Abbreviations: AMC, amoxicillin/clavulanic acid; AMI, aminoglycosides; AMP, ampicillin; BLBI, β-lactam beta-lactamase inhibitor combination; BPEN, benzylpenicillin; CAZ, ceftazidime, CG1, first-generation cephalosporins; CG3, third-generation cephalosporins; CIP, ciprofloxacin; CLI, clindamycin; CP, carbapenems; CRO, ceftriaxone; CZO, cefazolin; ERY, erythromycin; ETP, ertapenem; FL, fluoroquinolones; GEN, gentamicin; LI, lincosamides, LVX = levofloxacin; MEM, meropenem; ML, macrolides; PEN, penicillins; SU, sulfonamides; SXT, trimethoprim/sulfamethoxazole; TOB, tobramycin; TZP, piperacillin/tazobactam; VAN, vancomycin; X, other.

A complete listing of each of the 5 sets of HR estimates for each of the 110 pathogen–antibiotics and classification of their treatment relevance is provided in Supplement 1.3.

### Attributable Mortality Due to Resistance

When using the fitted models to predict the incidence of mortality in the presence of observed resistance patterns, as compared to the absence of resistance, attributable mortality due to antimicrobial resistance was 869 deaths (95% CI, 100–2555) in total over the 5-year study period, or 1.2 (95% CI, .7–17.6) per 100 000 population per year. Attributable mortality was distributed across all forms of bacteremia but was particularly present in bacteremia caused by *E. coli* (N = 265; 95% CI, 84–454), *E. faecium* (N = 102; 95% CI, −65 to 246), and *Klebsiella* spp. (N = 96; 95% CI, 20–185); complete results are provided in Supplement 1.4.

## DISCUSSION

In this study, we compared 30-day mortality in patients with bacteremia infected with susceptible versus resistant pathogens measuring the attributable impacts of AMR due to 110 pathogen–antibiotic combinations. We found that antimicrobial resistance was associated with a 10% relative increase in the risk of mortality among patients with bacteremia and 1.2 AMR attributable deaths per 100 000 population per year. The impacts of resistance were larger among antibiotics that were commonly used (for treating a given pathogen) and smaller in antibiotics that were not commonly used.

A recent study of the global impacts of AMR in 2019 estimated 16.4 global deaths attributable to AMR per 100 000 population, of which 4.8 per 100 000 were attributable to AMR bacteremia; in Canada, the estimates of death attributable to AMR overall and AMR bacteremia were 7.9 and 2.9 per 100 000 persons, respectively [17]. We found a moderately lower burden of resistance for AMR bacteremia, equal to 1.2 per 100 000 persons per year. A known issue in the AMR literature is underadjustment for confounding, in addition to selective or nonreporting of adjusted resistance effects [5]. Patients with resistant infections are generally older, frailer, and have had substantially longer healthcare exposures at the time of their index culture. A strength of our study was the robust adjustment for confounding by patient severity, as we were able to comprehensively adjust for multiple patient covariables including age, sex, comorbidities, and healthcare exposures, including ICU status, limiting the risk of residual confounding [18].

The mechanism driving associations between AMR and mortality remain unclear. Previous research suggests that there is an inconsistent and heterogeneous association between AMR and virulence that can be either positive or negative [19]. More likely, AMR increases mortality by impeding adequate treatment [5]. A systematic review of >70 studies suggested that there is a strong association between adequacy of antibiotic treatment and outcomes among patients with sepsis [20]. Studies also found important mortality impacts associated with resistance to first-line agents, known as difficult to treat resistance [21, 22]. We found that resistance to antibiotics commonly used in empiric treatment was associated with mortality whereas resistance to antibiotics not commonly used in empiric treatment was not, suggesting that adequacy, rather than virulence, is at play.

Our study has some notable limitations. First, we lacked complete resistance profiles for the specimens examined because our data were based on clinically reported susceptibilities. To address this, we imputed resistances that were masked, using a 2-step approach, first using a system of rules and then by imputing the probability of resistance using a logistic regression model [9]. Inaccurate imputation could have attenuated resistance associations; however, prior work suggests that results of analyses based on imputations are similar to those based on cases with complete resistance information [6]. The advantage of using clinical microbiology data is access to population-wide data linkable to important confounding and outcome data. Second, although we adjusted for an array of important comorbidities, residual confounding could have nevertheless impacted the results of our study. Third, we examined 30-day mortality rather than 60- or 90-day mortality because resistance effects in this shorter follow-up are likely larger; we suspect that use of a longer time frame could have further attenuated relative AMR associations. Finally, we lacked information on antibiotic regimens, both empiric and subsequent to blood cultures results, administered for inpatients, which could have helped verify treatment adequacy as an underlying mechanism.

Our study examined mortality associations for 110 forms of resistance among 30 bacteria and found that AMR is associated with 1.2 attributable deaths per 100 000 persons per year in Ontario, Canada. This rate of attributable mortality is lower than previous estimates, likely due to prior lack of adjustment for confounding by age, frailty, comorbidities, healthcare exposures, and co-resistance. The burden was principally due to resistance to antibiotics with empiric treatment relevance and was virtually zero for antibiotics with little empiric treatment relevance. These results suggest that treatment adequacy could be an important mechanism leading to AMR-associated mortality, and that the mortality burden will increase if resistance rates to our most common treatment agents increase. Strategies to limit the spread of AMR are crucial, and may already be working in North America, as indicated by decreasing rates of vancomycin-resistant enterococci, MRSA, and fluoroquinolone resistance in the Veterans Affairs administration of the United States, though these may be partly offset by increases in third-generation cephalosporin and carbapenem resistance [23]. Ongoing surveillance and global collaboration is needed to better document and limit the impacts of antimicrobial resistance.

## Supplementary Material

ciaf213_Supplementary_Data

## Supplementary Data

Supplementary materials are available at *Clinical Infectious Diseases* online. Consisting of data provided by the authors to benefit the reader, the posted materials are not copyedited and are the sole responsibility of the authors, so questions or comments should be addressed to the corresponding author.

## Notes

***Financial support**.* This study was funded by the Canadian Institutes of Health Research (grant number 401316).

***Potential conflicts of interest**.* The authors: No reported conflicts of interest. All authors have submitted the ICMJE Form for Disclosure of Potential Conflicts of Interest.
