# Supplementary material for: The Burden of Antimicrobial Resistant Bacteremia in Ontario: A Population-Wide Analysis of Attributable Mortality From 110 Pathogen-Antibiotic Combinations
Source: Clin Infect Dis. 2025 May 13;81(4):728–37. doi: 10.1093/cid/ciaf213 (PMC12596364; doi:10.1093/cid/ciaf213)
Supplement: ciaf213_Supplementary_Data [file ciaf213_supplementary_data.zip › TotalBurdenAMR_R1_Supplement1.docx]

# Supplement 1

[Supplement 1 1](#_Toc192157260)

[Supplement 1.1. Susceptibility and resistance imputation rules. 1](#_Toc192157261)

[Supplement 1.2. Treatment relevance of N=110 antibiotics 6](#_Toc192157262)

[Supplement 1.3. Antibiotic resistance hazard ratios (HRs) for 110 pathogen-antibiotic combinations. 7](#_Toc192157263)

[Supplement 1.4. Estimated 30-day mortality attributable to antibiotic resistance, by pathogen. 10](#_Toc192157264)

[Supplement 1.5. Code for running meta regression model and extraction of coefficients. 11](#_Toc192157265)

### Supplement 1.1. Susceptibility and resistance imputation rules.

| **pathogen** | **antibiotic** | **rule** |
| --- | --- | --- |
| Actinomyces spp. | AMIKACIN | Always R |
| Actinomyces spp. | AMOXICLAV | if penicillin S or ampicillin S then amoxiclav S |
| Actinomyces spp. | AMPICILLIN | if penicillin S, then ampicillin S. If Penicillin R, then Ampicillin R |
| Actinomyces spp. | CEFTRIAXONE | If Penicillin S, then Ceftriaxone S |
| Actinomyces spp. | ERTAPENEM | If Meropenem R, then Ertapenem R |
| Actinomyces spp. | GENTAMICIN | Always R |
| Actinomyces spp. | LEVOFLOXACIN | Always R |
| Actinomyces spp. | MOXIFLOXACIN | Always R |
| Actinomyces spp. | PENICILLIN | If Ampicllin S, then Penicillin S. |
| Actinomyces spp. | PIPTAZ | if penicillin S then piptaz S |
| Actinomyces spp. | TMP_SMX | Always R |
| Actinomyces spp. | TOBRAMYCIN | Always R |
| Clostridium spp. | AMIKACIN | Always R |
| Clostridium spp. | GENTAMICIN | Always R |
| Clostridium spp. | TMP_SMX | Always R |
| Clostridium spp. | TOBRAMYCIN | Always R |
| Clostridium spp. | VANCOMYCIN | Always R |
| Enterococcus faecium | AMIKACIN | Always R |
| Enterococcus faecium | AMOXICLAV | if ampicillin R, then R; if ampicillin S, then S |
| Enterococcus faecium | CEFAZOLIN | Always R |
| Enterococcus faecium | CEFTAZIDIME | Always R |
| Enterococcus faecium | CEFTRIAXONE | Always R |
| Enterococcus faecium | CIPROFLOXACIN | if levofloxacin or moxifloxacin available, assign the same result for ciprofloxacin |
| Enterococcus faecium | CLINDAMYCIN | Always R |
| Enterococcus faecium | CLOXACILLIN | Always R |
| Enterococcus faecium | DAPTOMYCIN | Always S |
| Enterococcus faecium | ERTAPENEM | Always R |
| Enterococcus faecium | GENTAMICIN | Always R |
| Enterococcus faecium | LEVOFLOXACIN | if ciprofloxacin R, then Rif ciprofloxacin or moxifloxacin available, assign the same result for levofloxacin |
| Enterococcus faecium | LINEZOLID | Always S |
| Enterococcus faecium | MEROPENEM | If Ampicilin S, then meropenem Sif ampicillin available then assign same result to meropenem |
| Enterococcus faecium | MOXIFLOXACIN | if ciprofloxacin R, then Rif ciprofloxacin or levofloxacin available, assign the same result for moxifloxacin |
| Enterococcus faecium | PIPTAZ | if ampicillin R, then R; if ampicillin S, then Sif ampicillin available then assign same result to piptaz |
| Enterococcus faecium | TMP_SMX | Always R |
| Enterococcus faecium | TOBRAMYCIN | Always R |
| Enterococcus spp., other | AMIKACIN | Always R |
| Enterococcus spp., other | AMOXICLAV | if ampicillin R, then R; if ampicillin S, then S |
| Enterococcus spp., other | CEFAZOLIN | Always R |
| Enterococcus spp., other | CEFTAZIDIME | Always R |
| Enterococcus spp., other | CEFTRIAXONE | Always R |
| Enterococcus spp., other | CLINDAMYCIN | Always R |
| Enterococcus spp., other | CLOXACILLIN | Always R |
| Enterococcus spp., other | DAPTOMYCIN | Always S |
| Enterococcus spp., other | ERTAPENEM | Always R |
| Enterococcus spp., other | GENTAMICIN | Always R |
| Enterococcus spp., other | LEVOFLOXACIN | if ciprofloxacin R, then R |
| Enterococcus spp., other | LINEZOLID | Always S |
| Enterococcus spp., other | MEROPENEM | If Ampicilin S, then meropenem Sif ampicillin available then assign same result to meropenem |
| Enterococcus spp., other | MOXIFLOXACIN | if ciprofloxacin R, then R |
| Enterococcus spp., other | PIPTAZ | if ampicillin R, then R; if ampicillin S, then Sif ampicillin available assign result to pip taz |
| Enterococcus spp., other | TMP_SMX | Always R |
| Enterococcus spp., other | TOBRAMYCIN | Always R |
| Escherichia coli | AMOXICLAV | If ceftriaxone or ceftazidime or meropenem or ertapenem R, then R; if ampicillin S then amoxiclav S |
| Escherichia coli | AMPICILLIN | if Cefazolin or Ceftriaxone or Ceftazidime or or Amox-clav or Pip-Tazo R or meropenem R or ertapenem R, then R |
| Escherichia coli | CEFAZOLIN | If Ceftriaxone R or Ceftazidime R or meropenem R or ertapenem R or piperacillin-tazobactam R, then R. if ampicillin S then cefazolin S; if ceftriaxone R then cefazolin R |
| Escherichia coli | CEFTAZIDIME | If meropenem R or ertapenem R, then R. if cefazolin S or ceftriaxone S then ceftazidime S |
| Escherichia coli | CEFTRIAXONE | if ampicillin S or cefazolin S, then S to ceftriaxone, If meropenem R or ertapenem R, then R. |
| Escherichia coli | CIPROFLOXACIN | if levofloxacin or moxifloxacin available, assign the same result for ciprofloxacin |
| Escherichia coli | CLINDAMYCIN | Always R |
| Escherichia coli | CLOXACILLIN | Always R |
| Escherichia coli | DAPTOMYCIN | Always R |
| Escherichia coli | ERTAPENEM | if cefazolin S or ceftriaxone S then ertapenem S; if meropenem R, then R to ertapenem |
| Escherichia coli | ERYTHROMYCIN | Always R |
| Escherichia coli | LEVOFLOXACIN | if ciprofloxacin R, then R. If ciprofloxacin S then S. if ciprofloxacin or moxifloxacin available, assign the same result for levofloxacin |
| Escherichia coli | LINEZOLID | Always R |
| Escherichia coli | MEROPENEM | If cefazolin S or ceftriaxone S or ertapenem S, then meropenem S . |
| Escherichia coli | MOXIFLOXACIN | if ciprofloxacin R, then Rif ciprofloxacin or levofloxacin available, assign the same result for moxifloxacin |
| Escherichia coli | PENICILLIN | Always R |
| Escherichia coli | PIPTAZ | If meropenem R or ertapenem R, then R. if cefazolin S or amoxiclav S then piptaz S |
| Escherichia coli | VANCOMYCIN | Always R |
| Granulicatella spp. | VANCOMYCIN | Always S |
| Morganella spp. | AMOXICLAV | Always R |
| Morganella spp. | AMPICILLIN | Always R |
| Morganella spp. | CEFAZOLIN | Always R |
| Morganella spp. | CIPROFLOXACIN | if levofloxacin or moxifloxacin available, assign the same result for ciprofloxacin |
| Morganella spp. | CLINDAMYCIN | Always R |
| Morganella spp. | CLOXACILLIN | Always R |
| Morganella spp. | DAPTOMYCIN | Always R |
| Morganella spp. | ERTAPENEM | if meropenem R, then ertapenem R |
| Morganella spp. | LEVOFLOXACIN | if ciprofloxacin R, then Rif ciprofloxacin or moxifloxacin available, assign the same result for levofloxacin |
| Morganella spp. | LINEZOLID | Always R |
| Morganella spp. | MEROPENEM | if ertapenem S, then meropenem S |
| Morganella spp. | MOXIFLOXACIN | if ciprofloxacin R, then Rif ciprofloxacin or levofloxacin available, assign the same result for moxifloxacin |
| Morganella spp. | PENICILLIN | Always R |
| Morganella spp. | VANCOMYCIN | Always R |
| Pseudomonas aeruginosa | AMOXICLAV | Always R |
| Pseudomonas aeruginosa | AMPICILLIN | Always R |
| Pseudomonas aeruginosa | CEFAZOLIN | Always R |
| Pseudomonas aeruginosa | CEFTRIAXONE | Always R |
| Pseudomonas aeruginosa | CLINDAMYCIN | Always R |
| Pseudomonas aeruginosa | CLOXACILLIN | Always R |
| Pseudomonas aeruginosa | DAPTOMYCIN | Always R |
| Pseudomonas aeruginosa | DOXYCYCLINE | Always R |
| Pseudomonas aeruginosa | ERTAPENEM | Always R |
| Pseudomonas aeruginosa | ERYTHROMYCIN | Always R |
| Pseudomonas aeruginosa | LEVOFLOXACIN | if ciprofloxacin R, then Rif ciprofloxacin R, assign levofloxacin R |
| Pseudomonas aeruginosa | LINEZOLID | Always R |
| Pseudomonas aeruginosa | MOXIFLOXACIN | Always R |
| Pseudomonas aeruginosa | PENICILLIN | Always R |
| Pseudomonas aeruginosa | TMP_SMX | Always R |
| Pseudomonas aeruginosa | VANCOMYCIN | Always R |
| Pseudomonas spp., other | AMPICILLIN | Always R |
| Pseudomonas spp., other | CEFAZOLIN | Always R |
| Pseudomonas spp., other | CEFTRIAXONE | Always R |
| Pseudomonas spp., other | CLINDAMYCIN | Always R |
| Pseudomonas spp., other | DAPTOMYCIN | Always R |
| Pseudomonas spp., other | ERYTHROMYCIN | Always R |
| Pseudomonas spp., other | LINEZOLID | Always R |
| Pseudomonas spp., other | PENICILLIN | Always R |
| Pseudomonas spp., other | VANCOMYCIN | Always R |
| Salmonella, non-typhoidal | AMIKACIN | Always R |
| Salmonella, non-typhoidal | CIPROFLOXACIN | if R to levofloxacin or moxifloxacin, assign R |
| Salmonella, non-typhoidal | CLINDAMYCIN | Always R |
| Salmonella, non-typhoidal | DAPTOMYCIN | Always R |
| Salmonella, non-typhoidal | GENTAMICIN | Always R |
| Salmonella, non-typhoidal | LEVOFLOXACIN | if R to ciprofloxacin or moxifloxacin, assign R |
| Salmonella, non-typhoidal | LINEZOLID | Always R |
| Salmonella, non-typhoidal | MOXIFLOXACIN | if R to ciprofloxacin or levofloxacin, assign R |
| Salmonella, non-typhoidal | TOBRAMYCIN | Always R |
| Salmonella, non-typhoidal | VANCOMYCIN | Always R |
| NA | AMIKACIN | Always R |
| NA | DAPTOMYCIN | Always R |
| NA | GENTAMICIN | Always R |
| NA | LINEZOLID | Always R |
| NA | TOBRAMYCIN | Always R |
| NA | VANCOMYCIN | Always R |
| Staphylococcus aureus | AMIKACIN | Always R |
| Staphylococcus aureus | AMOXICLAV | if cefazolin S, then S; if cloxacillin S, then S; if cefazolin R, then R, if cloxacillin R, then Rif cloxacillin or oxacillin or cefazolin available, assign the same result for amoxiclav |
| Staphylococcus aureus | AMPICILLIN | if Cefazolin R, then Rif penicillin available, then assign same result for ampicillin |
| Staphylococcus aureus | CEFAZOLIN | If cloxacillin S, then S; if cloxacillin R, then RIF cloxacillin-S then cefazolin S; if oxacillin S then Cefazolin S; If Cloxacillin-R then Cefazolin R, If oxacillin R then Cefazolin R |
| Staphylococcus aureus | CEFTAZIDIME | if cefazolin or cloxacillin or ceftriaxone R, then Rif R to cefazolin or oxacillin or cloxacillin, assign R to ceftazidime |
| Staphylococcus aureus | CEFTRIAXONE | if cefazolin or cloxacillin R, then RIF cloxacillin-S then ceftriaxone-S; if oxacillin S then Ceftraixone S; If Cefazolin S then Ceftriaxone S; If Cloxacillin-R then Cefazolin R, If oxacillin R then Cefazolin R; if Cefazolin R then Ceftriaxone R |
| Staphylococcus aureus | CIPROFLOXACIN | If levofloxacin or moxifloxacin R, then R |
| Staphylococcus aureus | CLOXACILLIN | if cefazolin S, then S; if cefazolin R, then RIf oxacillin or cefazolin available, assign cloxacillin the same result |
| Staphylococcus aureus | DAPTOMYCIN | Always S |
| Staphylococcus aureus | ERTAPENEM | if cefazolin S, then S; if cloxacillin S, then S; if cefazolin R, then R, if cloxacillin R, then R |
| Staphylococcus aureus | GENTAMICIN | Always R |
| Staphylococcus aureus | LEVOFLOXACIN | if moxifloxacin R, then R. If moxifloxacin S, then S. if ciprofloxacin S then levofloxacin S |
| Staphylococcus aureus | LINEZOLID | Always S |
| Staphylococcus aureus | MEROPENEM | if cefazolin S, then S; if cloxacillin S, then S; if cefazolin R, then R, if cloxacillin R, then Rif cloxacillin or oxacillin or cefazolin available, assign the same result for meropenem |
| Staphylococcus aureus | MOXIFLOXACIN | if levofloxacin R, then R. If levofloxacin S, then S. if ciprofloxacin or levofloxacin available, assign the same result for moxifloxacin |
| Staphylococcus aureus | PENICILLIN | if cefazolin or cloxacillin R, then R If ampicillin R or cefazolin R or oxacillin R or cloxacillin R, assign penicillin R |
| Staphylococcus aureus | PIPTAZ | if cefazolin or cloxacillin R, then RIF cloxacillin-S then piptaz-S; if oxacillin S then piptaz S; If Cefazolin S then Piptaz S; If Cloxacillin-R then Piptaz R, If oxacillin R then Piptaz R; if Cefazolin R then Piptaz R |
| Staphylococcus aureus | TOBRAMYCIN | Always R |
| Staphylococcus aureus | VANCOMYCIN | Always S |
| Staphylococcus epidermidis | AMOXICLAV | if cefazolin S, then S; if cloxacillin S, then S; if cefazolin R, then R, if cloxacillin R, then Rif cloxacillin or oxacillin or cefazolin or piptaz available, assign the same result for amoxiclav |
| Staphylococcus epidermidis | AMPICILLIN | if Cefazolin R, then Rif penicillin available, then assign same result for ampicillin |
| Staphylococcus epidermidis | CEFAZOLIN | If cloxacillin S, then S; if cloxacillin R, then Rif oxacillin or cloxacillin available, assign cefazolin the same result |
| Staphylococcus epidermidis | CEFTAZIDIME | if cefazolin or cloxacillin or ceftriaxone R, then Rif cloxacillin or oxacillin or cefazolin are R, then R for ceftaz |
| Staphylococcus epidermidis | CEFTRIAXONE | if cefazolin or cloxacillin R, then Rif cloxacillin or oxacillin or cefazolin available, assign the same result for ceftriaxone |
| Staphylococcus epidermidis | CIPROFLOXACIN | if levofloxacin or moxifloxacin available, assign the same result for ciprofloxacin |
| Staphylococcus epidermidis | CLOXACILLIN | If oxacillin or cefazolin available, assign cloxacillin the same result |
| Staphylococcus epidermidis | DAPTOMYCIN | Always S |
| Staphylococcus epidermidis | ERTAPENEM | if cefazolin S, then S; if cloxacillin S, then S; if cefazolin R, then R, if cloxacillin R, then R |
| Staphylococcus epidermidis | GENTAMICIN | Always R |
| Staphylococcus epidermidis | LEVOFLOXACIN | if moxifloxacin R, then R |
| Staphylococcus epidermidis | LINEZOLID | Always S |
| Staphylococcus epidermidis | MEROPENEM | if cefazolin S, then S; if cloxacillin S, then S; if cefazolin R, then R, if cloxacillin R, then Rif cloxacillin or oxacillin or cefazolin or piptaz or amoxiclav or ertapenem available, assign the same result for mero |
| Staphylococcus epidermidis | MOXIFLOXACIN | if levofloxacin R, then Rif ciprofloxacin or levofloxacin available, assign the same result for moxifloxacin |
| Staphylococcus epidermidis | PIPTAZ | if cefazolin or oxacillin available, assign same result for piptaz |
| Staphylococcus epidermidis | TOBRAMYCIN | Always R |
| Staphylococcus epidermidis | VANCOMYCIN | Always S |
| Staphylococcus lugdunensis | AMIKACIN | Always R |
| Staphylococcus lugdunensis | AMOXICLAV | if cefazolin S, then S; if cloxacillin S, then S; if cefazolin R, then R, if cloxacillin R, then Rif cloxacillin or oxacillin or cefazolin available, assign the same result for amoxiclav |
| Staphylococcus lugdunensis | CEFAZOLIN | If cloxacillin S, then S; if cloxacillin R, then Rif oxacillin or cloxacillin available, assign cefazolin the same result |
| Staphylococcus lugdunensis | CEFTAZIDIME | if cefazolin or cloxacillin or ceftriaxone R, then R |
| Staphylococcus lugdunensis | CEFTRIAXONE | if cefazolin or cloxacillin R, then Rif cloxacillin or oxacillin or cefazolin available, assign the same result for ceftriaxone |
| Staphylococcus lugdunensis | CIPROFLOXACIN | if levofloxacin or moxifloxacin available, assign the same result for ciprofloxacin |
| Staphylococcus lugdunensis | CLOXACILLIN | if cefazolin S, then S; if cefazolin R, then RIf oxacillin or cefazolin available, assign cloxacillin the same result |
| Staphylococcus lugdunensis | DAPTOMYCIN | Always S |
| Staphylococcus lugdunensis | ERTAPENEM | if cefazolin S, then S; if cloxacillin S, then S; if cefazolin R, then R, if cloxacillin R, then R |
| Staphylococcus lugdunensis | GENTAMICIN | Always R |
| Staphylococcus lugdunensis | LEVOFLOXACIN | if moxifloxacin R, then R |
| Staphylococcus lugdunensis | LINEZOLID | Always S |
| Staphylococcus lugdunensis | MEROPENEM | if cefazolin S, then S; if cloxacillin S, then S; if cefazolin R, then R, if cloxacillin R, then Rif cloxacillin or oxacillin or cefazolin available, assign the same result for meropenem |
| Staphylococcus lugdunensis | MOXIFLOXACIN | if levofloxacin R, then R |
| Staphylococcus lugdunensis | PIPTAZ | if cefazolin or cloxacillin R, then Rif cloxacillin or oxacillin or cefazolin available, assign the same result for piptaz |
| Staphylococcus lugdunensis | TOBRAMYCIN | Always R |
| Staphylococcus lugdunensis | VANCOMYCIN | Always S |
| Staphylococcus spp., other | AMIKACIN | Always R |
| Staphylococcus spp., other | AMOXICLAV | if cefazolin S, then S; if cloxacillin S, then S; if cefazolin R, then R, if cloxacillin R, then Rif cloxacillin or oxacillin or cefazolin available, assign the same result for amoxiclav |
| Staphylococcus spp., other | AMPICILLIN | if Cefazolin R, then Rif R to cefazolin or oxacillin or cloxacillin, assign R to ampicillin |
| Staphylococcus spp., other | CEFAZOLIN | If cloxacillin S, then S; if cloxacillin R, then Rif oxacillin or cloxacillin available, assign cefazolin the same result |
| Staphylococcus spp., other | CEFTAZIDIME | if cefazolin or cloxacillin or ceftriaxone R, then Rif cloxacillin or oxacillin or cefazolin are R, then R for ceftaz |
| Staphylococcus spp., other | CEFTRIAXONE | if cefazolin or cloxacillin R, then Rif cloxacillin or oxacillin or cefazolin available, assign the same result for ceftriaxone |
| Staphylococcus spp., other | CIPROFLOXACIN | If levofloxacin or moxifloxacin R, then Rif levofloxacin or moxifloxacin available, assign the same result for ciprofloxacin |
| Staphylococcus spp., other | CLOXACILLIN | if cefazolin S, then S; if cefazolin R, then RIf oxacillin or cefazolin available, assign cloxacillin the same result |
| Staphylococcus spp., other | DAPTOMYCIN | Always S |
| Staphylococcus spp., other | ERTAPENEM | if cefazolin S, then S; if cloxacillin S, then S; if cefazolin R, then R, if cloxacillin R, then R |
| Staphylococcus spp., other | GENTAMICIN | Always R |
| Staphylococcus spp., other | LEVOFLOXACIN | if moxifloxacin R, then R. If moxifloxacin S, then S. if ciprofloxacin or moxifloxacin available, assign the same result for levofloxacin |
| Staphylococcus spp., other | LINEZOLID | Always S |
| Staphylococcus spp., other | MEROPENEM | if cefazolin S, then S; if cloxacillin S, then S; if cefazolin R, then R, if cloxacillin R, then Rif cloxacillin or oxacillin or cefazolin available, assign the same result for meropenem |
| Staphylococcus spp., other | MOXIFLOXACIN | if levofloxacin R, then R. If levofloxacin S, then S. if ciprofloxacin or levofloxacin available, assign the same result for moxifloxacin |
| Staphylococcus spp., other | PENICILLIN | if cefazolin or cloxacillin R, then R |
| Staphylococcus spp., other | PIPTAZ | if cefazolin or cloxacillin R, then Rif cloxacillin or oxacillin or cefazolin available, assign the same result for piptaz |
| Staphylococcus spp., other | TOBRAMYCIN | Always R |
| Staphylococcus spp., other | VANCOMYCIN | Always S |
| Streptococcus viridians spp. | AMIKACIN | Always R |
| Streptococcus viridians spp. | AMOXICLAV | if penicillin S then amoxiclav S |
| Streptococcus viridians spp. | AMPICILLIN | if penicillin S then ampicillin S |
| Streptococcus viridians spp. | CEFAZOLIN | if penicillin S then cefazolin S |
| Streptococcus viridians spp. | CEFTRIAXONE | If Penicillin S, then Ceftriaxone S |
| Streptococcus viridians spp. | CIPROFLOXACIN | if levofloxacin or moxifloxacin available, assign the same result for ciprofloxacin |
| Streptococcus viridians spp. | CLOXACILLIN | if penicillin S then cloxacillin S |
| Streptococcus viridians spp. | DAPTOMYCIN | Always S |
| Streptococcus viridians spp. | ERTAPENEM | if penicillin S then ertapenem S |
| Streptococcus viridians spp. | GENTAMICIN | Always R |
| Streptococcus viridians spp. | LEVOFLOXACIN | if moxifloxacin R, then R |
| Streptococcus viridians spp. | LINEZOLID | Always S |
| Streptococcus viridians spp. | MEROPENEM | if penicillin S then meropenem S |
| Streptococcus viridians spp. | MOXIFLOXACIN | if ciprofloxacin or levofloxacin available, assign the same result for moxifloxacin |
| Streptococcus viridians spp. | PENICILLIN | if ceftriaxone S then penicillin S |
| Streptococcus viridians spp. | PIPTAZ | if penicillin S then piptaz S |
| Streptococcus viridians spp. | VANCOMYCIN | Always S |
| Streptococcus group B | AMIKACIN | Always R |
| Streptococcus group B | AMOXICLAV | Always S |
| Streptococcus group B | AMPICILLIN | Always S |
| Streptococcus group B | CEFAZOLIN | Always S |
| Streptococcus group B | CEFTRIAXONE | Always S |
| Streptococcus group B | CIPROFLOXACIN | if levofloxacin or moxifloxacin available, assign the same result for ciprofloxacin |
| Streptococcus group B | CLOXACILLIN | Always S |
| Streptococcus group B | DAPTOMYCIN | Always S |
| Streptococcus group B | ERTAPENEM | Always S |
| Streptococcus group B | GENTAMICIN | Always R |
| Streptococcus group B | LEVOFLOXACIN | if moxifloxacin R, then R |
| Streptococcus group B | LINEZOLID | Always S |
| Streptococcus group B | MEROPENEM | Always S |
| Streptococcus group B | PENICILLIN | Always S |
| Streptococcus group B | PIPTAZ | Always S |
| Streptococcus group B | TOBRAMYCIN | Always R |
| Streptococcus group B | VANCOMYCIN | Always S |
| Streptococcus group C/G | AMPICILLIN | Always S |
| Streptococcus group C/G | CEFAZOLIN | Always S |
| Streptococcus group C/G | CEFTRIAXONE | Always S |
| Streptococcus group C/G | CIPROFLOXACIN | if levofloxacin or moxifloxacin available, assign the same result for ciprofloxacin |
| Streptococcus group C/G | CLOXACILLIN | Always S |
| Streptococcus group C/G | DAPTOMYCIN | Always S |
| Streptococcus group C/G | GENTAMICIN | Always R |
| Streptococcus group C/G | LINEZOLID | Always S |
| Streptococcus group C/G | MEROPENEM | Always S |
| Streptococcus group C/G | PENICILLIN | Always S |
| Streptococcus group C/G | PIPTAZ | Always S |
| Streptococcus group C/G | TOBRAMYCIN | Always R |
| Streptococcus group C/G | VANCOMYCIN | Always S |
| Streptococcus mitis | AMIKACIN | Always R |
| Streptococcus mitis | DAPTOMYCIN | Always S |
| Streptococcus mitis | GENTAMICIN | Always R |
| Streptococcus mitis | LINEZOLID | Always S |
| Streptococcus mitis | TOBRAMYCIN | Always R |
| Streptococcus mitis | VANCOMYCIN | Always S |
| Streptococcus pneumoniae | AMIKACIN | Always R |
| Streptococcus pneumoniae | AMOXICLAV | if penicillin S or ampicillin S then amoxiclav S |
| Streptococcus pneumoniae | AMPICILLIN | if penicillin S then ampicillin S |
| Streptococcus pneumoniae | CEFAZOLIN | if penicillin S then cefazolin S |
| Streptococcus pneumoniae | CEFTRIAXONE | If Penicillin S, then Ceftriaxone S |
| Streptococcus pneumoniae | CLOXACILLIN | if penicillin S then cloxacillin S |
| Streptococcus pneumoniae | DAPTOMYCIN | Always S |
| Streptococcus pneumoniae | ERTAPENEM | if penicillin S then ertapenem S |
| Streptococcus pneumoniae | GENTAMICIN | Always R |
| Streptococcus pneumoniae | LEVOFLOXACIN | if moxifloxacin R, then R |
| Streptococcus pneumoniae | LINEZOLID | Always S |
| Streptococcus pneumoniae | MEROPENEM | if penicillin S then meropenem S |
| Streptococcus pneumoniae | MOXIFLOXACIN | if levofloxacin R, then Rif levofloxacin available, assign the same result for moxifloxacin |
| Streptococcus pneumoniae | PENICILLIN | if ceftriaxone S then penicillin S |
| Streptococcus pneumoniae | PIPTAZ | if penicillin S then piptaz S |
| Streptococcus pneumoniae | TOBRAMYCIN | Always R |
| Streptococcus pneumoniae | VANCOMYCIN | Always S |
| Streptococcus pyogenes | AMIKACIN | Always R |
| Streptococcus pyogenes | AMOXICLAV | Always S |
| Streptococcus pyogenes | AMPICILLIN | Always S |
| Streptococcus pyogenes | CEFAZOLIN | Always S |
| Streptococcus pyogenes | CEFTRIAXONE | Always S |
| Streptococcus pyogenes | CLOXACILLIN | Always S |
| Streptococcus pyogenes | DAPTOMYCIN | Always S |
| Streptococcus pyogenes | ERTAPENEM | Always S |
| Streptococcus pyogenes | GENTAMICIN | Always R |
| Streptococcus pyogenes | LEVOFLOXACIN | if moxifloxacin R, then R |
| Streptococcus pyogenes | LINEZOLID | Always S |
| Streptococcus pyogenes | MEROPENEM | Always S |
| Streptococcus pyogenes | PENICILLIN | Always S |
| Streptococcus pyogenes | PIPTAZ | Always S |
| Streptococcus pyogenes | TOBRAMYCIN | Always R |
| Streptococcus pyogenes | VANCOMYCIN | Always S |
| Streptococcus spp., other | AMIKACIN | Always R |
| Streptococcus spp., other | CEFTRIAXONE | If Penicillin S, then Ceftriaxone Sif penicillin S then ceftriaxone S |
| Streptococcus spp., other | DAPTOMYCIN | Always S |
| Streptococcus spp., other | GENTAMICIN | Always R |
| Streptococcus spp., other | LEVOFLOXACIN | if moxifloxacin R, then R |
| Streptococcus spp., other | LINEZOLID | Always S |
| Streptococcus spp., other | MOXIFLOXACIN | if levlofloxacin R, then R |
| Streptococcus spp., other | TOBRAMYCIN | Always R |
| Streptococcus spp., other | VANCOMYCIN | Always S |

### Supplement 1.2. Treatment relevance of N=110 antibiotics

Based on adjudication by 2 study authors (DM and KS). Adjudicators were blinded to the study outcomes. Each adjudicator rated each antibiotic’s relevance for each pathogen, on a 3-point scale (low=0, moderate=0.5, high=1), and the average of the 2 ratings was taken, yielding a 5-point scale (0=low, 0.25=moderate-low, 0.5=moderate, 0.75=moderate-high, 1=high).

| **Antibiotic class** |  | **AMI** | **AMI** | **PEN** | **PEN** | **BLBI** | **BLBI** | **CG1** | **CG3** | **CG3** | **CP** | **CP** | **FL** | **FL** | **LI** | **ML** | **SU** | **X** |
| --- | --- | --- | --- | --- | --- | --- | --- | --- | --- | --- | --- | --- | --- | --- | --- | --- | --- | --- |
| **Antibiotic name** |  | **GEN** | **TOB** | **AMP** | **PEN** | **AMC** | **TZP** | **CZO** | **CAZ** | **CRO** | **ETP** | **MEM** | **CIP** | **LVX** | **CLI** | **ERY** | **SXT** | **VAN** |
|  | N |  |  |  |  |  |  |  |  |  |  |  |  |  |  |  |  |  |
| **Gram Negative Bacteria** |  |  |  |  |  |  |  |  |  |  |  |  |  |  |  |  |  |  |
| *Enterobacterales* |  |  |  |  |  |  |  |  |  |  |  |  |  |  |  |  |  |  |
| Escherichia coli | 25,928 | 0.25 |  | 0.50 |  | 0.75 | 1.00 | 0.50 |  | 1.00 |  |  | 0.75 |  |  |  | 0.75 |  |
| Klebsiella spp. | 8,382 | 0.25 |  |  |  | 0.75 | 1.00 | 0.50 |  | 1.00 |  |  | 0.75 |  |  |  | 0.75 |  |
| Enterobacter spp. | 1,975 | 0.25 |  |  |  |  | 0.75 |  |  | 0.75 | 1.00 | 1.00 | 0.75 |  |  |  | 0.75 |  |
| Proteus spp. | 1,847 | 0.25 |  | 0.50 |  | 0.75 | 1.00 | 0.50 |  | 1.00 |  |  | 0.75 |  |  |  | 0.75 |  |
| Serratia spp. | 925 |  |  |  |  |  | 1.00 |  |  | 1.00 | 1.00 |  | 0.75 |  |  |  |  |  |
| Citrobacter spp. | 622 | 0.25 |  |  |  |  | 0.75 | 0.25 |  | 0.75 | 1.00 |  | 0.75 |  |  |  | 0.75 |  |
| Salmonella, non-typhoidal | 425 |  |  | 0.50 |  |  |  |  |  |  |  |  | 1.00 |  |  |  |  |  |
| Morganella spp. | 285 | 0.25 |  |  |  |  | 1.00 |  |  | 1.00 |  |  | 0.75 |  |  |  | 0.75 |  |
| *Non-fermenters* |  |  |  |  |  |  |  |  |  |  |  |  |  |  |  |  |  |  |
| Pseudomonas aeruginosa | 3,082 |  | 0.25 |  |  |  | 1.00 |  | 1.00 |  |  | 1.00 | 0.75 |  |  |  |  |  |
| Acinetobacter spp. | 573 | 0.25 |  |  |  |  | 1.00 |  | 1.00 |  |  | 1.00 | 0.75 |  |  |  | 0.75 |  |
| Stenotrophomonas spp. | 290 |  |  |  |  |  |  |  |  |  |  |  |  | 0.25 |  |  |  |  |
| Pseudomonas spp., other | 267 |  |  |  |  |  | 1.00 |  |  |  |  |  | 0.75 |  |  |  | 0.25 |  |
| *Other* |  |  |  |  |  |  |  |  |  |  |  |  |  |  |  |  |  |  |
| Hemophilus spp. | 548 |  |  | 0.50 |  |  |  |  |  |  |  |  |  |  |  |  |  |  |
| Bacteroides spp. | 477 |  |  |  |  |  | 1.00 |  |  |  |  |  |  |  | 0.25 |  |  |  |
| **Gram Positive Bacteria** |  |  |  |  |  |  |  |  |  |  |  |  |  |  |  |  |  |  |
| *Staphylococcus* |  |  |  |  |  |  |  |  |  |  |  |  |  |  |  |  |  |  |
| Staphylococcus aureus | 14,854 |  |  |  | 0.00 |  |  | 1.00 |  |  |  |  |  |  | 0.25 | 0.00 | 0.50 |  |
| Staphylococcus spp., other | 5,240 |  |  |  | 0.00 |  |  | 1.00 |  |  |  |  |  |  | 0.25 | 0.00 | 0.50 |  |
| Staphylococcus epidermidis | 3,693 |  |  |  | 0.00 |  |  | 0.50 |  |  |  |  |  |  | 0.25 | 0.00 | 0.50 |  |
| Staphylococcus lugdunensis | 419 |  |  |  | 0.00 |  |  | 1.00 |  |  |  |  |  |  |  |  |  |  |
| *Streptococcus* |  |  |  |  |  |  |  |  |  |  |  |  |  |  |  |  |  |  |
| Streptococcus spp., other | 2,993 |  |  |  | 1.00 |  |  |  |  | 1.00 |  |  |  |  | 0.25 |  |  |  |
| Streptococcus pneumoniae | 2,332 |  |  |  | 1.00 |  |  |  |  |  |  |  |  |  | 0.00 | 0.25 | 0.25 |  |
| Streptococcus pyogenes | 1,612 |  |  |  |  |  |  |  |  |  |  |  |  |  | 0.50 | 0.00 |  |  |
| Streptococcus group B | 1,606 |  |  |  |  |  |  |  |  |  |  |  |  |  | 0.50 | 0.00 |  |  |
| Streptococcus group C/G | 1,299 |  |  |  |  |  |  |  |  |  |  |  |  |  | 0.25 | 0.00 |  |  |
| Streptococcus viridians spp. | 943 |  |  |  | 1.00 |  |  |  |  | 1.00 |  |  |  |  |  |  |  |  |
| Streptococcus mitis | 612 |  |  |  | 1.00 |  |  |  |  | 1.00 |  |  |  |  |  |  |  |  |
| *Enterococcus* |  |  |  |  |  |  |  |  |  |  |  |  |  |  |  |  |  |  |
| Enterococcus faecium | 1,738 |  |  | 0.75 |  |  |  |  |  |  |  |  |  |  |  |  |  | 1.00 |
| Enterococcus spp., other* | 299 |  |  | 1.00 |  |  |  |  |  |  |  |  |  |  |  |  |  | 1.00 |
| *Other* |  |  |  |  |  |  |  |  |  |  |  |  |  |  |  |  |  |  |
| Clostridium spp. | 341 |  |  |  | 0.50 |  | 1.00 |  |  |  |  |  |  |  | 0.50 |  |  |  |
| Actinomyces spp. | 203 |  |  |  |  |  |  |  |  |  |  |  |  |  | 0.00 |  |  |  |
| Granulicatella spp. | 152 |  |  |  | 1.00 |  |  |  |  | 1.00 |  |  |  |  |  |  |  |  |

Abbreviations: Antibiotics – GEN = Gentamicin, TOB = Tobramycin, AMP = Ampicillin, PEN = Benzylpenicillin, AMC = Amoxicillin/clavulanic acid, TZP = Piperacillin/tazobactam, CZO = Cefazolin, CAZ = Ceftazidime, CRO = Ceftriaxone, ETP = Ertapenem, MEM = Meropenem, CIP = Ciprofloxacin, LVX = Levofloxacin, CLI = Clindamycin, ERY = Erythromycin, SXT = Trimethoprim/sulfamethoxazole, VAN = Vancomycin; antibiotic classes – AMI = aminoglycosides, PEN = penicillins, BLBI = beta-lactam beta-lactamase inhibitor combination, CG1 = 1^st^ generation cephalosporins, CG3 = 3^rd^ generation cephalosporins, CP = carbapenems, FL = fluoroquinolones, LI = lincosamides, ML = macrolides, SU = sulfonamides, X = other.

### Supplement 1.3. Antibiotic resistance hazard ratios (HRs) for 110 pathogen-antibiotic combinations.

Based on 5 regression models.

| **Pathogen** | **Treat** | **Antibiotic** | **Unadjusted**  **HR** | **Covariate**  **adjusted HR** | **Co-resistance adjusted HR** | **Covariate & co-resistance**  **adjusted HR** | **Covariate & co-resistance**  **adjusted HR based on meta-regression** |
| --- | --- | --- | --- | --- | --- | --- | --- |
| Acinetobacter spp. | 1 | ceftazidime | 2.09 (0.74,5.85) | 2.92 (1.04,8.16) | 1.45 (0.45,4.69) | 1.91 (0.61,5.99) | 1.22 (0.38,3.96) |
| Acinetobacter spp. | 0.75 | ciprofloxacin | 1.54 (0.33,7.30) | 1.13 (0.24,5.38) | 0.12 (0.01,1.95) | 0.09 (0.01,1.41) | 1.18 (0.07,19.41) |
| Acinetobacter spp. | 0.25 | gentamicin | 4.42 (1.02,19.12) | 6.26 (1.44,27.20) | 3.68 (0.46,29.49) | 5.51 (0.66,45.82) | 1.07 (0.13,8.71) |
| Acinetobacter spp. | 1 | meropenem | 8.95 (1.35,59.43) | 18.10 (2.80,99.99) | 23.25 (1.68,99.99) | 31.32 (1.89,99.99) | 1.26 (0.08,21.12) |
| Acinetobacter spp. | 1 | piptaz | 1.40 (0.49,4.03) | 1.47 (0.52,4.18) | 0.92 (0.29,2.94) | 0.93 (0.30,2.87) | 1.19 (0.39,3.61) |
| Acinetobacter spp. | 0.75 | tmp_smx | 4.37 (0.99,19.26) | 2.21 (0.51,9.69) | 2.70 (0.46,15.78) | 1.21 (0.19,7.63) | 1.20 (0.18,8.10) |
| Actinomyces spp. | 0 | clindamycin | 2.53 (0.52,12.33) | 1.80 (0.41,8.02) | 2.55 (0.53,12.30) | 1.81 (0.41,7.89) | 0.97 (0.22,4.22) |
| Bacteroides spp. | 0.25 | clindamycin | 1.86 (1.01,3.42) | 1.50 (0.83,2.70) | 1.90 (1.03,3.51) | 1.59 (0.88,2.87) | 1.11 (0.59,2.10) |
| Bacteroides spp. | 1 | piptaz | 0.96 (0.39,2.39) | 0.78 (0.31,1.97) | 1.12 (0.45,2.77) | 1.06 (0.44,2.50) | 1.19 (0.48,2.94) |
| Citrobacter spp. | 0.25 | cefazolin | 8.31 (0.87,79.00) | 7.02 (0.77,64.11) | 6.92 (0.72,66.07) | 5.88 (0.64,54.06) | 1.09 (0.12,9.77) |
| Citrobacter spp. | 0.75 | ceftriaxone | 2.16 (0.97,4.78) | 2.31 (1.03,5.16) | 1.75 (0.62,4.98) | 1.97 (0.70,5.55) | 1.20 (0.43,3.34) |
| Citrobacter spp. | 0.75 | ciprofloxacin | 0.68 (0.14,3.45) | 0.62 (0.12,3.12) | 1.09 (0.18,6.44) | 0.97 (0.15,6.44) | 1.17 (0.19,7.42) |
| Citrobacter spp. | 1 | ertapenem | 10.93 (2.26,52.84) | 6.11 (1.14,32.69) | 6.25 (1.17,33.28) | 3.75 (0.64,21.96) | 1.19 (0.20,6.91) |
| Citrobacter spp. | 0.25 | gentamicin | 0.97 (0.16,5.95) | 1.20 (0.20,7.25) | 1.09 (0.09,13.10) | 1.38 (0.11,17.77) | 1.00 (0.08,12.91) |
| Citrobacter spp. | 0.75 | piptaz | 3.00 (1.44,6.23) | 3.64 (1.72,7.68) | 1.52 (0.53,4.32) | 1.52 (0.54,4.32) | 1.18 (0.40,3.43) |
| Citrobacter spp. | 0.75 | tmp_smx | 0.29 (0.06,1.38) | 0.39 (0.08,1.85) | 0.31 (0.04,2.37) | 0.33 (0.04,2.63) | 1.09 (0.14,8.32) |
| Clostridium spp. | 0.5 | clindamycin | 0.81 (0.37,1.81) | 0.95 (0.43,2.10) | 0.80 (0.35,1.80) | 0.87 (0.38,1.96) | 1.09 (0.47,2.53) |
| Clostridium spp. | 0.5 | penicillin | 0.99 (0.40,2.45) | 1.39 (0.56,3.48) | 1.19 (0.47,3.01) | 1.66 (0.64,4.30) | 1.11 (0.42,2.96) |
| Clostridium spp. | 1 | piptaz | 1.05 (0.05,23.45) | 1.00 (0.04,22.61) | 1.02 (0.04,24.50) | 0.78 (0.03,19.04) | 1.18 (0.05,29.49) |
| Enterobacter spp. | 0.75 | ceftriaxone | 1.20 (0.87,1.66) | 1.19 (0.86,1.64) | 0.82 (0.46,1.47) | 0.93 (0.52,1.64) | 1.14 (0.61,2.16) |
| Enterobacter spp. | 0.75 | ciprofloxacin | 1.31 (0.75,2.29) | 1.18 (0.68,2.07) | 1.07 (0.57,2.02) | 1.02 (0.54,1.94) | 1.13 (0.56,2.27) |
| Enterobacter spp. | 1 | ertapenem | 1.22 (0.88,1.68) | 1.22 (0.88,1.68) | 1.83 (1.06,3.16) | 1.55 (0.90,2.68) | 1.25 (0.68,2.29) |
| Enterobacter spp. | 0.25 | gentamicin | 1.73 (0.81,3.69) | 1.34 (0.62,2.88) | 1.55 (0.63,3.82) | 1.33 (0.53,3.31) | 1.04 (0.41,2.68) |
| Enterobacter spp. | 1 | meropenem | 3.59 (1.12,11.47) | 3.21 (0.99,10.39) | 1.75 (0.49,6.24) | 1.90 (0.53,6.82) | 1.26 (0.33,4.77) |
| Enterobacter spp. | 0.75 | piptaz | 1.45 (1.00,2.09) | 1.53 (1.06,2.22) | 1.63 (0.94,2.84) | 1.69 (0.97,2.95) | 1.20 (0.64,2.23) |
| Enterobacter spp. | 0.75 | tmp_smx | 1.36 (0.83,2.23) | 1.20 (0.73,1.98) | 1.07 (0.57,2.01) | 0.99 (0.52,1.86) | 1.10 (0.57,2.15) |
| Enterococcus faecium | 0.75 | ampicillin | 2.84 (1.76,4.56) | 2.67 (1.68,4.23) | 2.88 (1.75,4.74) | 2.73 (1.68,4.44) | 1.37 (0.77,2.43) |
| Enterococcus faecium | 1 | vancomycin | 1.13 (0.87,1.46) | 1.11 (0.86,1.43) | 1.00 (0.77,1.30) | 1.00 (0.77,1.28) | 1.14 (0.85,1.54) |
| Enterococcus spp., other | 1 | ampicillin | 1.80 (0.78,4.16) | 2.00 (0.81,4.94) | 1.75 (0.76,4.03) | 2.04 (0.84,4.94) | 1.24 (0.51,3.06) |
| Enterococcus spp., other | 1 | vancomycin | 0.60 (0.29,1.23) | 0.56 (0.27,1.15) | 0.61 (0.30,1.26) | 0.56 (0.27,1.14) | 1.11 (0.53,2.35) |
| Escherichia coli | 0.75 | amoxiclav | 1.66 (1.46,1.88) | 1.28 (1.13,1.45) | 1.02 (0.71,1.45) | 0.81 (0.57,1.15) | 1.01 (0.68,1.50) |
| Escherichia coli | 0.5 | ampicillin | 1.39 (1.24,1.57) | 1.20 (1.06,1.34) | 0.97 (0.80,1.18) | 1.03 (0.85,1.25) | 1.01 (0.80,1.26) |
| Escherichia coli | 0.5 | cefazolin | 1.48 (1.32,1.67) | 1.23 (1.10,1.39) | 1.09 (0.91,1.31) | 1.06 (0.88,1.26) | 1.04 (0.84,1.29) |
| Escherichia coli | 1 | ceftriaxone | 1.92 (1.65,2.24) | 1.40 (1.20,1.63) | 1.32 (0.93,1.87) | 1.32 (0.94,1.85) | 1.17 (0.80,1.71) |
| Escherichia coli | 0.75 | ciprofloxacin | 1.86 (1.64,2.10) | 1.32 (1.17,1.50) | 1.55 (1.33,1.80) | 1.19 (1.02,1.38) | 1.16 (0.95,1.41) |
| Escherichia coli | 0.25 | gentamicin | 1.33 (1.11,1.60) | 1.10 (0.91,1.31) | 0.91 (0.75,1.11) | 0.91 (0.75,1.11) | 0.96 (0.76,1.23) |
| Escherichia coli | 1 | piptaz | 2.12 (1.82,2.46) | 1.50 (1.29,1.74) | 1.19 (0.91,1.56) | 1.22 (0.94,1.59) | 1.18 (0.86,1.61) |
| Escherichia coli | 0.75 | tmp_smx | 1.35 (1.19,1.55) | 1.20 (1.05,1.36) | 1.04 (0.90,1.21) | 1.07 (0.92,1.23) | 1.08 (0.90,1.31) |
| Granulicatella spp. | 1 | ceftriaxone | 1.23 (0.36,4.24) | 1.99 (0.50,7.94) | 1.61 (0.43,5.96) | 2.81 (0.62,12.74) | 1.19 (0.26,5.48) |
| Granulicatella spp. | 1 | penicillin | 0.49 (0.13,1.86) | 0.49 (0.12,2.02) | 0.42 (0.10,1.75) | 0.35 (0.07,1.68) | 1.18 (0.25,5.64) |
| Hemophilus spp. | 0.5 | ampicillin | 1.54 (0.65,3.62) | 1.29 (0.56,2.94) | 1.53 (0.65,3.62) | 1.29 (0.56,2.94) | 1.10 (0.46,2.62) |
| Klebsiella spp. | 0.75 | amoxiclav | 1.81 (1.47,2.23) | 1.44 (1.16,1.78) | 1.44 (0.94,2.21) | 1.32 (0.86,2.02) | 1.19 (0.74,1.89) |
| Klebsiella spp. | 0.5 | cefazolin | 1.36 (1.15,1.62) | 1.18 (0.99,1.40) | 1.06 (0.85,1.33) | 0.97 (0.78,1.20) | 1.03 (0.79,1.35) |
| Klebsiella spp. | 1 | ceftriaxone | 2.24 (1.77,2.85) | 1.85 (1.45,2.36) | 1.57 (0.97,2.54) | 1.66 (1.03,2.69) | 1.26 (0.76,2.11) |
| Klebsiella spp. | 0.75 | ciprofloxacin | 1.51 (1.16,1.96) | 1.34 (1.03,1.75) | 0.95 (0.67,1.34) | 0.97 (0.69,1.37) | 1.08 (0.73,1.59) |
| Klebsiella spp. | 0.25 | gentamicin | 2.18 (1.50,3.18) | 1.76 (1.21,2.57) | 1.38 (0.87,2.19) | 1.28 (0.81,2.03) | 1.07 (0.65,1.76) |
| Klebsiella spp. | 1 | piptaz | 2.06 (1.61,2.63) | 1.60 (1.24,2.06) | 0.95 (0.62,1.44) | 0.83 (0.55,1.27) | 1.12 (0.70,1.78) |
| Klebsiella spp. | 0.75 | tmp_smx | 1.44 (1.11,1.85) | 1.25 (0.96,1.61) | 1.00 (0.72,1.39) | 0.96 (0.69,1.33) | 1.08 (0.74,1.57) |
| Morganella spp. | 1 | ceftriaxone | 2.48 (0.84,7.30) | 2.89 (0.97,8.64) | 1.32 (0.31,5.57) | 1.83 (0.41,8.08) | 1.21 (0.27,5.42) |
| Morganella spp. | 0.75 | ciprofloxacin | 2.12 (0.67,6.72) | 3.11 (0.85,11.38) | 1.21 (0.25,6.02) | 1.99 (0.45,8.85) | 1.14 (0.26,5.04) |
| Morganella spp. | 0.25 | gentamicin | 3.59 (0.96,13.49) | 3.14 (0.69,14.24) | 1.42 (0.22,9.15) | 0.66 (0.10,4.14) | 1.04 (0.16,6.87) |
| Morganella spp. | 1 | piptaz | 3.25 (1.14,9.28) | 3.44 (1.20,9.85) | 2.97 (0.71,12.42) | 2.50 (0.60,10.45) | 1.24 (0.29,5.30) |
| Morganella spp. | 0.75 | tmp_smx | 1.81 (0.62,5.28) | 2.17 (0.67,7.04) | 1.29 (0.33,5.07) | 1.90 (0.53,6.86) | 1.13 (0.32,4.05) |
| Proteus spp. | 0.75 | amoxiclav | 1.43 (0.91,2.23) | 1.49 (0.96,2.32) | 0.70 (0.21,2.34) | 0.68 (0.21,2.27) | 1.07 (0.32,3.61) |
| Proteus spp. | 0.5 | ampicillin | 1.18 (0.84,1.67) | 1.20 (0.85,1.69) | 1.49 (0.95,2.34) | 1.38 (0.89,2.15) | 1.11 (0.69,1.78) |
| Proteus spp. | 0.5 | cefazolin | 1.14 (0.83,1.56) | 1.20 (0.88,1.64) | 1.02 (0.71,1.46) | 1.10 (0.78,1.56) | 1.07 (0.72,1.57) |
| Proteus spp. | 1 | ceftriaxone | 1.17 (0.52,2.60) | 1.43 (0.65,3.16) | 1.16 (0.24,5.60) | 1.43 (0.30,6.81) | 1.21 (0.25,5.83) |
| Proteus spp. | 0.75 | ciprofloxacin | 1.35 (0.87,2.08) | 1.10 (0.71,1.72) | 1.65 (1.00,2.73) | 1.22 (0.74,2.01) | 1.11 (0.65,1.90) |
| Proteus spp. | 0.25 | gentamicin | 1.17 (0.62,2.19) | 1.28 (0.69,2.38) | 1.05 (0.52,2.13) | 1.20 (0.60,2.41) | 1.04 (0.50,2.18) |
| Proteus spp. | 1 | piptaz | 2.47 (0.91,6.71) | 2.36 (0.88,6.34) | 1.87 (0.34,10.32) | 1.60 (0.29,8.64) | 1.23 (0.22,6.87) |
| Proteus spp. | 0.75 | tmp_smx | 0.80 (0.50,1.30) | 0.90 (0.56,1.43) | 0.51 (0.28,0.92) | 0.64 (0.36,1.13) | 1.05 (0.58,1.89) |
| Pseudomonas aeruginosa | 1 | ceftazidime | 2.44 (1.72,3.46) | 1.87 (1.31,2.68) | 3.12 (1.84,5.29) | 2.22 (1.33,3.72) | 1.31 (0.74,2.31) |
| Pseudomonas aeruginosa | 0.75 | ciprofloxacin | 0.98 (0.66,1.44) | 0.98 (0.67,1.45) | 0.69 (0.45,1.05) | 0.77 (0.50,1.17) | 1.05 (0.66,1.67) |
| Pseudomonas aeruginosa | 1 | meropenem | 1.98 (1.31,2.97) | 1.47 (0.93,2.33) | 1.74 (1.06,2.86) | 1.50 (0.91,2.49) | 1.24 (0.73,2.12) |
| Pseudomonas aeruginosa | 1 | piptaz | 1.71 (1.19,2.47) | 1.47 (1.02,2.13) | 0.70 (0.40,1.22) | 0.81 (0.47,1.39) | 1.19 (0.67,2.11) |
| Pseudomonas aeruginosa | 0.25 | tobramycin | 1.76 (0.66,4.74) | 1.62 (0.61,4.35) | 2.00 (0.73,5.46) | 2.07 (0.77,5.53) | 1.08 (0.40,2.93) |
| Pseudomonas spp. | 0.75 | ciprofloxacin | 2.14 (0.38,12.01) | 1.70 (0.31,9.23) | 1.98 (0.36,10.89) | 1.83 (0.37,9.08) | 1.14 (0.22,5.82) |
| Pseudomonas spp. | 1 | piptaz | 2.82 (0.85,9.36) | 1.75 (0.54,5.68) | 3.86 (1.10,13.50) | 1.66 (0.51,5.44) | 1.23 (0.38,4.01) |
| Pseudomonas spp. | 0.25 | tmp_smx | 1.19 (0.45,3.20) | 1.94 (0.74,5.09) | 0.84 (0.30,2.33) | 1.42 (0.55,3.62) | 1.09 (0.42,2.87) |
| Salmonella, non-typhoidal | 0.5 | ampicillin | 0.32 (0.01,10.58) | 0.58 (0.03,9.86) | 2.24 (0.08,60.28) | 1.82 (0.12,28.10) | 0.98 (0.06,15.42) |
| Salmonella, non-typhoidal | 1 | ciprofloxacin | 0.61 (0.11,3.43) | 0.92 (0.23,3.71) | 0.65 (0.11,3.73) | 0.94 (0.23,3.82) | 0.99 (0.24,4.11) |
| Serratia spp. | 1 | ceftriaxone | 1.53 (0.83,2.83) | 1.66 (0.91,3.01) | 1.44 (0.65,3.18) | 1.70 (0.80,3.63) | 1.23 (0.56,2.71) |
| Serratia spp. | 0.75 | ciprofloxacin | 1.26 (0.40,4.04) | 1.80 (0.59,5.47) | 1.14 (0.36,3.68) | 1.69 (0.55,5.14) | 1.14 (0.37,3.51) |
| Serratia spp. | 1 | ertapenem | 2.64 (0.42,16.50) | 4.03 (0.67,24.13) | 3.65 (0.57,23.50) | 4.30 (0.72,25.57) | 1.25 (0.20,7.82) |
| Serratia spp. | 1 | piptaz | 1.55 (0.90,2.67) | 1.50 (0.89,2.56) | 1.33 (0.61,2.89) | 1.06 (0.50,2.26) | 1.19 (0.56,2.55) |
| Staphylococcus aureus | 1 | cefazolin | 1.11 (0.96,1.27) | 1.13 (0.98,1.30) | 1.11 (0.96,1.28) | 1.12 (0.97,1.29) | 1.14 (0.95,1.37) |
| Staphylococcus aureus | 0.25 | clindamycin | 1.25 (1.08,1.45) | 1.09 (0.95,1.26) | 1.47 (1.13,1.92) | 1.09 (0.84,1.41) | 1.04 (0.77,1.39) |
| Staphylococcus aureus | 0 | erythromycin | 1.21 (1.04,1.40) | 1.16 (1.00,1.34) | 0.76 (0.54,1.06) | 0.90 (0.66,1.24) | 0.96 (0.67,1.37) |
| Staphylococcus aureus | 0 | penicillin | 0.97 (0.62,1.53) | 1.21 (0.77,1.90) | 0.89 (0.54,1.47) | 0.97 (0.60,1.57) | 0.97 (0.59,1.60) |
| Staphylococcus aureus | 0.5 | tmp_smx | 0.07 (0.03,0.20) | 1.17 (0.62,2.22) | 0.48 (0.24,0.99) | 1.02 (0.51,2.03) | 1.08 (0.53,2.20) |
| Staphylococcus epidermidis | 0.5 | cefazolin | 1.61 (1.27,2.04) | 1.31 (1.03,1.67) | 1.49 (1.16,1.92) | 1.24 (0.97,1.60) | 1.15 (0.85,1.57) |
| Staphylococcus epidermidis | 0.25 | clindamycin | 1.89 (1.35,2.65) | 1.44 (1.02,2.05) | 0.93 (0.58,1.48) | 0.94 (0.59,1.49) | 1.02 (0.62,1.68) |
| Staphylococcus epidermidis | 0 | erythromycin | 1.29 (0.86,1.95) | 0.99 (0.65,1.50) | 0.97 (0.59,1.62) | 0.95 (0.57,1.59) | 0.98 (0.56,1.72) |
| Staphylococcus epidermidis | 0 | penicillin | 1.12 (0.54,2.31) | 1.57 (0.75,3.26) | 0.77 (0.27,2.13) | 1.10 (0.40,3.04) | 1.00 (0.35,2.82) |
| Staphylococcus epidermidis | 0.5 | tmp_smx | 2.39 (1.78,3.21) | 1.52 (1.13,2.05) | 1.61 (1.15,2.24) | 1.34 (0.96,1.87) | 1.16 (0.78,1.71) |
| Staphylococcus lugdunensis | 1 | cefazolin | 0.26 (0.04,1.52) | 0.44 (0.07,2.77) | 0.26 (0.04,1.56) | 0.47 (0.07,3.01) | 1.18 (0.19,7.34) |
| Staphylococcus lugdunensis | 0 | penicillin | 0.45 (0.08,2.45) | 0.20 (0.04,1.11) | 0.37 (0.06,2.17) | 0.28 (0.05,1.61) | 0.96 (0.16,5.82) |
| Staphylococcus spp. Other | 1 | cefazolin | 1.63 (1.36,1.95) | 1.28 (1.06,1.54) | 1.43 (1.17,1.76) | 1.16 (0.95,1.43) | 1.20 (0.93,1.55) |
| Staphylococcus spp., other | 0.25 | clindamycin | 2.30 (1.71,3.10) | 1.49 (1.08,2.04) | 0.83 (0.51,1.36) | 0.79 (0.48,1.32) | 0.99 (0.57,1.72) |
| Staphylococcus spp., other | 0 | erythromycin | 0.71 (0.52,0.97) | 1.29 (0.93,1.78) | 0.82 (0.50,1.33) | 1.06 (0.65,1.74) | 0.98 (0.57,1.69) |
| Staphylococcus spp., other | 0 | penicillin | 1.71 (1.01,2.91) | 1.30 (0.77,2.21) | 1.24 (0.65,2.37) | 1.07 (0.56,2.03) | 0.99 (0.50,1.98) |
| Staphylococcus spp., other | 0.5 | tmp_smx | 2.30 (1.84,2.89) | 1.73 (1.38,2.17) | 1.81 (1.39,2.36) | 1.57 (1.22,2.03) | 1.26 (0.92,1.75) |
| Stenotrophomonas spp. | 0.25 | levofloxacin | 6.32 (1.92,20.72) | 6.56 (2.12,20.29) | 11.46 (3.30,39.78) | 13.00 (4.12,41.04) | 1.18 (0.35,3.91) |
| Streptococcus viridians spp. | 1 | ceftriaxone | 0.21 (0.01,3.15) | 0.16 (0.01,2.46) | 0.36 (0.02,5.65) | 0.28 (0.02,4.37) | 1.15 (0.07,18.69) |
| Streptococcus viridians spp. | 1 | penicillin | 0.47 (0.24,0.91) | 0.46 (0.24,0.89) | 0.49 (0.25,0.97) | 0.50 (0.25,0.98) | 1.07 (0.52,2.20) |
| Streptococcus group C/G | 0.25 | clindamycin | 0.26 (0.10,0.66) | 0.39 (0.15,0.97) | 0.23 (0.07,0.76) | 0.28 (0.08,0.97) | 1.00 (0.29,3.44) |
| Streptococcus group C/G | 0 | erythromycin | 0.25 (0.09,0.67) | 0.39 (0.15,1.00) | 1.46 (0.41,5.26) | 1.63 (0.43,6.19) | 0.94 (0.24,3.69) |
| Streptococcus group B | 0.5 | clindamycin | 0.99 (0.64,1.56) | 1.07 (0.68,1.66) | 0.67 (0.37,1.20) | 0.69 (0.38,1.23) | 1.07 (0.57,2.00) |
| Streptococcus group B | 0 | erythromycin | 1.31 (0.85,2.04) | 1.40 (0.91,2.16) | 2.22 (1.07,4.60) | 2.31 (1.13,4.74) | 1.05 (0.49,2.23) |
| Streptococcus mitis | 1 | ceftriaxone | 1.84 (0.40,8.59) | 2.04 (0.43,9.71) | 3.27 (0.60,17.67) | 2.58 (0.48,13.82) | 1.22 (0.21,7.02) |
| Streptococcus mitis | 1 | penicillin | 0.63 (0.29,1.35) | 0.83 (0.39,1.77) | 0.53 (0.23,1.22) | 0.74 (0.33,1.66) | 1.15 (0.49,2.70) |
| Streptococcus pneumoniae | 0 | clindamycin | 1.79 (0.53,5.99) | 0.61 (0.14,2.67) | 0.49 (0.08,2.93) | 0.40 (0.06,2.56) | 0.96 (0.14,6.51) |
| Streptococcus pneumoniae | 0.25 | erythromycin | 2.20 (0.99,4.88) | 1.24 (0.54,2.84) | 1.46 (0.52,4.09) | 1.16 (0.42,3.22) | 1.03 (0.36,2.93) |
| Streptococcus pneumoniae | 1 | penicillin | 2.30 (1.06,5.00) | 1.67 (0.79,3.53) | 2.36 (1.08,5.19) | 1.73 (0.81,3.69) | 1.24 (0.54,2.83) |
| Streptococcus pneumoniae | 0.25 | tmp_smx | 0.54 (0.22,1.33) | 0.78 (0.32,1.93) | 0.46 (0.14,1.50) | 0.56 (0.17,1.83) | 1.01 (0.30,3.36) |
| Streptococcus pyogenes | 0.5 | clindamycin | 0.40 (0.14,1.10) | 0.90 (0.34,2.35) | 0.48 (0.13,1.72) | 0.95 (0.30,3.07) | 0.99 (0.30,3.26) |
| Streptococcus pyogenes | 0 | erythromycin | 0.30 (0.10,0.94) | 0.63 (0.21,1.90) | 0.69 (0.13,3.72) | 0.75 (0.16,3.66) | 0.95 (0.20,4.55) |
| Streptococcus spp., other | 1 | ceftriaxone | 0.42 (0.08,2.07) | 0.78 (0.17,3.50) | 0.65 (0.13,3.09) | 0.90 (0.19,4.18) | 1.17 (0.26,5.33) |
| Streptococcus spp., other | 0.25 | clindamycin | 0.32 (0.12,0.87) | 0.58 (0.23,1.45) | 0.38 (0.13,1.15) | 0.40 (0.13,1.18) | 0.98 (0.32,3.04) |
| Streptococcus spp., other | 1 | penicillin | 0.76 (0.50,1.16) | 0.95 (0.63,1.44) | 0.73 (0.44,1.22) | 0.88 (0.53,1.46) | 1.13 (0.65,1.97) |

### Supplement 1.4. Estimated 30-day mortality attributable to antibiotic resistance, by pathogen.

| **Pathogen** | **Attributable 30-day**  **Mortality, N (95% CI)** |
| --- | --- |
| Escherichia coli | 265 (84, 454) |
| Klebsiella spp. | 96 (20, 185) |
| Enterobacter spp. | 60 (-18, 143) |
| Proteus spp. | 23 (-18, 82) |
| Serratia spp. | 29 (-39, 86) |
| Citrobacter spp. | 20 (-219, 107) |
| Salmonella, non-typhoidal | -1 (-22, 47) |
| Morganella spp. | 9 (-56, 91) |
| Pseudomonas aeruginosa | 42 (-3, 93) |
| Acinetobacter spp. | 7 (-8, 32) |
| Stenotrophomonas spp. | 3 (-8, 15) |
| Hemophilus spp. | 1 (-8, 12) |
| Bacteroides spp. | 8 (-33, 58) |
| Staphylococcus aureus | 10 (-1144, 890) |
| Staphylococcus spp., other | 100 (-321, 396) |
| Staphylococcus epidermidis | 71 (-473, 382) |
| Staphylococcus lugdunensis | -2 (-89, 68) |
| Streptococcus spp., other | 4 (-66, 76) |
| Streptococcus pneumoniae | 3 (-99, 107) |
| Streptococcus pyogenes | -1 (-18, 26) |
| Streptococcus group B | 7 (-32, 45) |
| Streptococcus group C/G | -1 (-21, 27) |
| Streptococcus viridians spp. | 2 (-15, 29) |
| Streptococcus mitis | 2 (-8, 16) |
| Enterococcus faecium | 102 (-65, 246) |
| Enterococcus spp., other | 4 (-17, 26) |
| Clostridium spp. | 3 (-13, 20) |
| Actinomyces spp. | -0 (-7, 9) |
| Granulicatella spp. | 3 (-19, 32) |

### Supplement 1.5. Further description and code for running meta regression model and extraction of coefficients.

library(brms)

2 datasets were used for the multivariate meta-regression. The first (d1) has rows corresponding to each log hazard ratio from the 30 pathogen-specific Weibull regression models (30*1 scales + 30*1 intercepts + 30*11 risk factors + 110 rows for the antibiotics). This came to 500 rows. The dataset has organism, variable name, and variable type as columns, in addition to the log hazard ratios. The second (d2) is a square matrix with 500 rows and 500 columns that has variances and covariances corresponding to each of the coefficients in d1. For example, the value in row 1, column 1 of d2 is the variance of the 1^st^ row of table d1 (i.e. the variance of age, in the Acinetobacter model). The value in position row 1, column 2 of d2 is the covariance of the coefficient in position 1 with position 2 (i.e. covariance of age with ceftazidime, in the Acinetobacter model). So all diagonal elements represent variances will off diagonals represent covariances. Covariance values are only non-zero within a given organism.

d1<-read_xlsx("TotalBurdenAMR_Supplement2.xlsx",1)

d2<-read_xlsx("TotalBurdenAMR_Supplement2.xlsx",2)

d1$organism_variable <- paste0(d1$organism,"-", d1$variable)

The code below runs the meta regression model.

fo <- bf(loghr ~ 0 + variable_type + treat +

(1| gr(organism_variable, by=variable_type)) + fcor(vcov))

m <- brm(fo, chains=8, cores = 8, iter=10, data=d1, data2=list(vcov=d2),backend="cmdstanr")

The code below extracts the estimated partially pooled coefficients and their 95% confidence intervals, from the meta regression model.

m95<-function(x) { out<-c(mean(x),quantile(x,.025),quantile(x,.975)); names(out)<-c("mean","p025","p975");out }

draws_coefs <- as_draws_df(m) %>% tibble()

draws_coefs$b_abxtrt<-draws_coefs$b_variable_typeabx+draws_coefs$b_treat

m_coeftable<-apply(draws_coefs,2,function(x) m95(x)) %>% t() %>% data.frame() %>% rownames_to_column(var = "coef") %>% tibble()

m_coeftable
